# Harmonic Generation up to Fifth Order from Al/Au/CuS Nanoparticle Films

**DOI:** 10.1021/acs.nanolett.4c00776

**Published:** 2024-04-15

**Authors:** Yueming Yan, Nathan J. Spear, Qingzhou Meng, Mahi R. Singh, Janet E. Macdonald, Richard F. Haglund

**Affiliations:** † Department of Physics and Astronomy, Vanderbilt University, Nashville, Tennessee 37235, United States; ‡ Interdisciplinary Materials Science, Vanderbilt University, Nashville, Tennessee 37235, United States; § Department of Chemistry, Vanderbilt Institute of Nanoscale Science and Engineering, Vanderbilt University, Nashville, Tennessee 37235, United States; ∥ Department of Physics and Astronomy, The University of Western Ontario, London N6A 3K7, Canada

**Keywords:** fifth-harmonic generation, fourth-harmonic generation, multiplasmonic triple-layer nanostructures, cascaded upconversion, plasmonic enhancement, aluminum nanoparticles

## Abstract

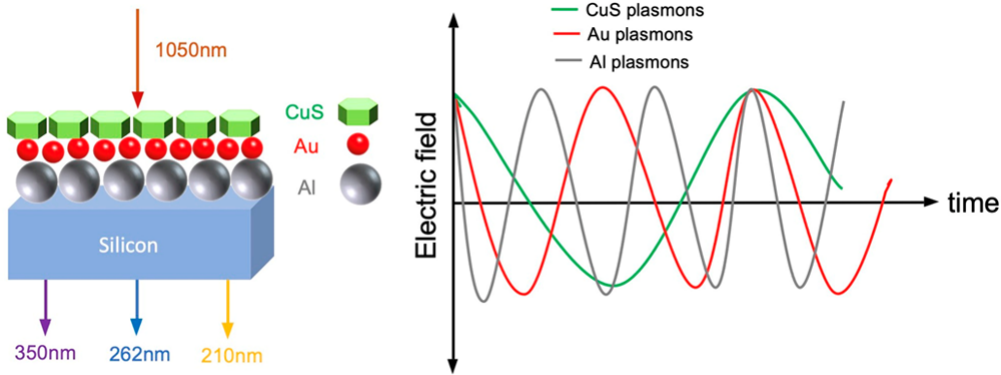

Dual heterostructures integrating noble-metal and copper chalcogenide nanoparticles have attracted a great deal of attention in nonlinear optics, because coupling of their localized surface plasmon resonances (LSPRs) substantially enhances light–matter interactions through local-field effects. Previously, enhanced cascaded third-harmonic generation was demonstrated in Au/CuS heterostructures mediated by harmonically coupled surface plasmon resonances. This suggests a promising approach for extending nonlinear enhancement to higher harmonics by adding an additional nanoparticulate material with higher-frequency harmonic resonances to the hybrid films. Here we report the first observation of enhanced cascaded fourth- and fifth-harmonic generation in Al/Au/CuS driven by coupled LSPRs at the fundamental (1050 nm), second harmonic (525 nm), and third harmonic (350 nm) of the pump frequency. An analytical model based on incoherent dipole–dipole interactions among plasmonic nanoparticles accounts for the observed enhancements. The results suggest a novel design for efficiently generating higher harmonics in resonant plasmonic structures by means of multiple sum-frequency cascades.

Plasmonic materials employing localized surface plasmon resonances (LSPRs) are attractive for applications ranging from photovoltaics^1−3^ to biomedical sensing^4−6^ and biomedical imaging.^7−9^ Most studies of plasmonic optical properties focus on either noble metals (gold,^10^ silver,^11^ and copper^12^) or heavily doped semiconductors, such as copper chalcogenides^13,14^ and tungsten oxides,^15,16^ in which LSPRs originate from coherent collective oscillations of charge carriers driven by the electric field of incident light. Because LSPRs depend strongly on nanostructure shape, size, and composition as well as the dielectric function of the embedding matrix, plasmonic nanoparticles (NPs) could be tunable from the ultraviolet (UV) to the near infrared (NIR). The charge-carrier oscillations can induce extremely large local electric-field enhancements near nanoparticle surfaces, which strongly enhance the nonlinear optical response, as, for instance, in surface-enhanced Raman scattering,^17−19^ harmonic generation,^20−23^ and multiphoton photoluminescence (MPPL).^24−26^

Recently, significantly enhanced nonlinear optical effects due to plasmon–plasmon coupling^27−29^ were observed in plasmonic heterostructures comprising noble-metal and semiconductor NPs. These heterostructures typically have distinct surface plasmon bands from the metal and semiconductor, respectively: the noble-metal LSPRs are designed to match a harmonic of the semiconductor plasmon. When the fundamental plasmon resonance in the semiconductor is excited, synergistic plasmon–plasmon interactions intensify the light–matter interaction and stimulate even more efficient upconversion or harmonic generation than a single-nanoparticle system. Also, such composite nanostructures lead to improved performance in photocatalysis,^30,31^ solar energy conversion,^32,33^ and photothermal and photodynamic cancer therapy.^34,35^

While gold and silver NPs have attracted the most attention for nanoplasmonics, aluminum NPs also present intriguing opportunities. First, aluminum is abundant and inexpensive compared to noble metals. Second, the strong interband transition in Al between parallel bands around the ∑ axis on the Γ–K–W–X plane is localized in a narrow energy range around 1.5 eV.^36^ Only for excitation energies within this narrow energy interval will aluminum plasmons decay rapidly into electron–hole pairs by interband damping. For excitation energies below or above this energy (as in our case for a pump laser at 1.18 eV), interband coupling is weak, and Al NPs will support long-lived LSPRs. Third, the free-electron density of aluminum is higher than that of either gold or silver, as is the plasma frequency, so that Al LSPRs can be tuned deeply in the UV.^37−39^ The aluminum nanocrystals studied here feature ultraviolet LSPRs at 350 nm, corresponding to the third-harmonic condition of the pump photon energy.

Previously, we reported 3-fold enhanced second-harmonic generation (SHG)^29^ and 20-fold enhanced third-harmonic generation (THG)^40^ from the Au/CuS heterostructure films compared with the incoherent sum of the SHG/THG yield from constituent NPs. The enhanced THG arose from a cascade process in which second-harmonic photons generated from CuS plasmons would be transported to Au plasmons and then yield the third-harmonic light by sum-frequency generation (SFG) sequentially with another fundamental photon from the incident light. The process of high-efficiency cascaded THG, mediated by the fundamental and second-harmonic surface plasmon resonance, motivates this exploration of higher-order-harmonic generation by establishing a multiplasmonic structure with the plasmon resonance at higher harmonics. We predict that the structure with the resonance at the fundamental, second harmonic, and third harmonics could lead to the further buildup of cascaded fourth-harmonic and fifth-harmonic generation (4HG and 5HG, respectively).

Here, we optimize the facile synthesis of high-purity monodisperse aluminum nanocrystals from the literature.^41^ Because Al LSPRs depend strongly on size, we control the nanocrystal diameter, making Al NPs with LSPRs at the blue-shifted value of 280 nm and red-shifted values of 375 and 350 nm exactly matching the third-harmonic frequency of the 1050 nm incident light, respectively. Then a triple-layer heterostructure containing combinations of Al, Au, and CuS films is formed that demonstrated a 6-fold 5HG enhancement and a 7-fold 4HG enhancement compared with those of the Au/CuS hybrid films. Additionally, the disappearance of enhanced harmonic generation in systems containing the aluminum NPs whose LSPRs are shifted from 350 nm further suggests the critical effect of the harmonic condition of the surface plasmon resonances. As both a conceptual and experimental extension of the previous study, the enhanced fourth- and fifth-harmonic signals are assumed to be produced in a cascade of several second-order SFG processes. Finally, an analytical model is developed for the nanohybrid made of an ensemble of Al, Au, and CuS NPs. With the additional contribution from the electric field induced by the surface plasmon polariton and dipole–dipole interaction, the 4HG and 5HG enhancements are both reproduced theoretically. The consistency between the theoretical calculations and experimental results strongly implies the cascaded mechanisms of higher-order-harmonic generation.

Aluminum NPs are synthesized following an optimized protocol based on the literature^41^ and confirmed by X-ray diffraction (XRD). The XRD pattern of the Al NPs is shown in Figure S1, highlighting the most characteristic diffraction peaks of the fcc form of aluminum. Also presented are transmission electron microscopy (TEM) images with increasing ratios of tetrahydrofuran to 1,4-dioxane in the reaction solution, the result being that the average diameter of the spherical Al NPs increased from 98 to 122 nm and then to 147 nm (Figure 1a–c). Additionally, the size-dependent extinction spectra of aluminum NPs are characterized by UV–visible–NIR spectrophotometry. Figure S3 shows that the 122 nm diameter aluminum NPs exhibit the desired dipolar plasmon resonance at 350 nm (the third harmonic of the pump laser). As the diameter increases to 147 nm, the resonance peak red-shifts to 375 nm; when the diameter decreases to 98 nm, the resonance peak blue-shifts to 280 nm.

**Figure 1:**
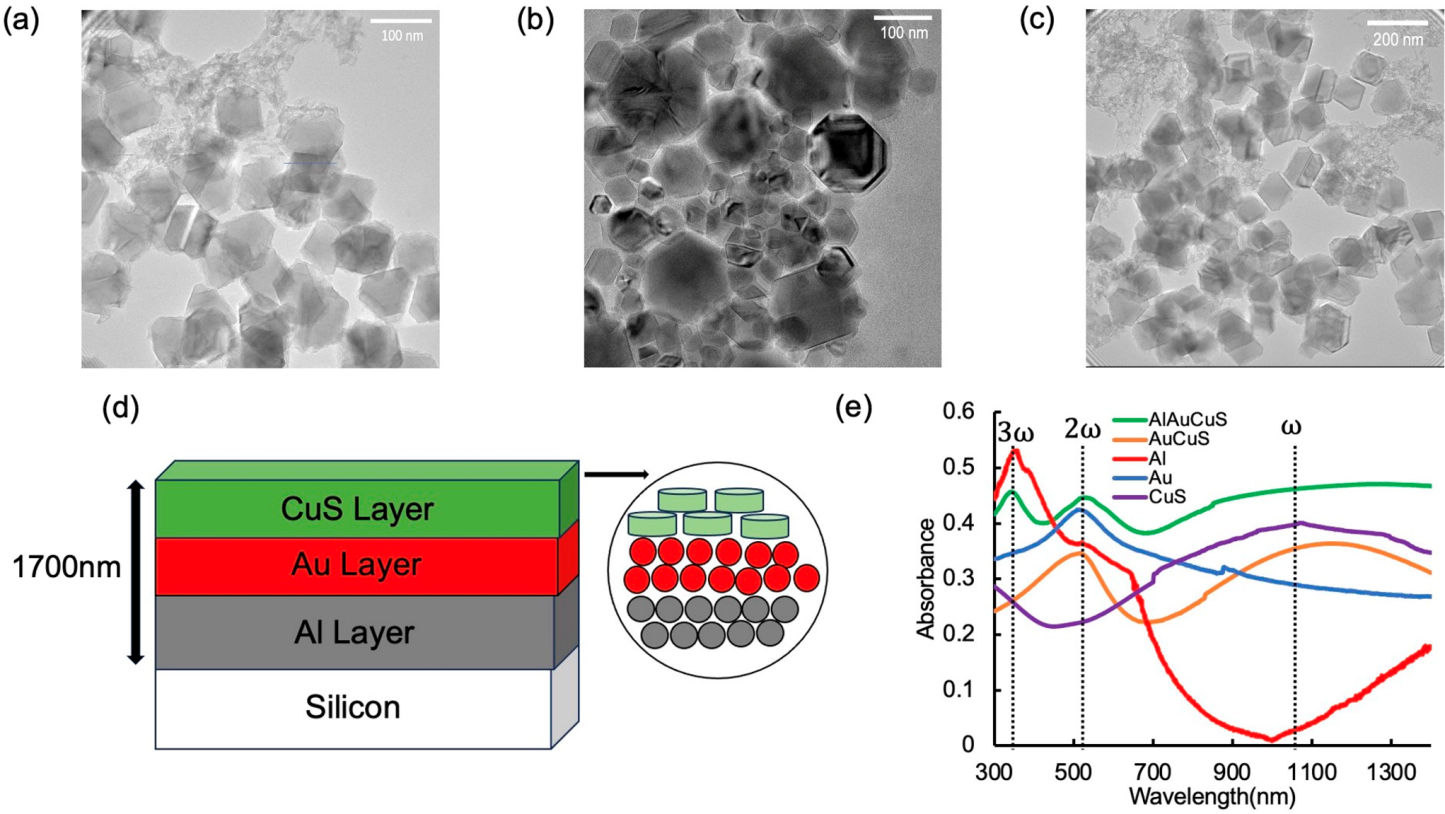
TEM images of aluminum nanoparticles with average diameters of (a) 98 ± 17 nm (*n* = 44), (b) 122 ± 20 nm (*n* = 30), and (c) 147 ± 14 nm (*n* = 48). (d) Schematic diagram of AlAuCuS heterostructure films. (e) UV–visible–NIR spectra of typical nanoparticle films. Here the heterostructure films contain 122 nm Al nanoparticles.

Then Al, Au, and CuS NPs are deposited sequentially onto a microscope slide by spin coating, thus assembling into a triple-layer heterostructure (Figure 1d). The thickness of the combined films is measured by profilometry to be ∼1700 nm (Figure S2). Bilayer Al–CuS and Au–CuS films are similarly prepared. The UV–visible–NIR spectra of the Al/Au/CuS films exhibit three distinct extinction peaks, which are from the LSPRs of CuS (1050 nm), Au (525 nm), and Al (350 nm) (Figure 1e). Per a previous investigation,^40^ we assume that the harmonic relationship among the three LSPRs is a prerequisite for plasmonic interactions that generate enhanced harmonics in the heterostructure upon irradiation with 1050 nm light.

The idler pulse train (S1 Optical measurements) of an Orpheus-F optical parametric amplifier at 1050 nm is used to excite the samples. Third-, fourth-, and fifth- harmonic signals for Al/Au/CuS, Al/CuS, Au/CuS, Al, Au, and CuS films are collected by a UV-sensitive photomultiplier tube with appropriate band-pass filters. Plots of the 5HG, 4HG, and THG intensities as a function of incident pump intensity are shown in panels a–c, respectively, of Figure 2. The power dependence of the harmonic intensity on the pump intensity at 1050 nm is obtained from log–log plots (Figure 2c–e). The slope and *R*^2^ values of the linear log–log fits for the harmonic generation data are included in Table 1. The slopes of the intensity–pump power curves are close to 3, 4, and 5 for the third, fourth, and fifth harmonic signals, respectively, from these films.

**Figure 2:**
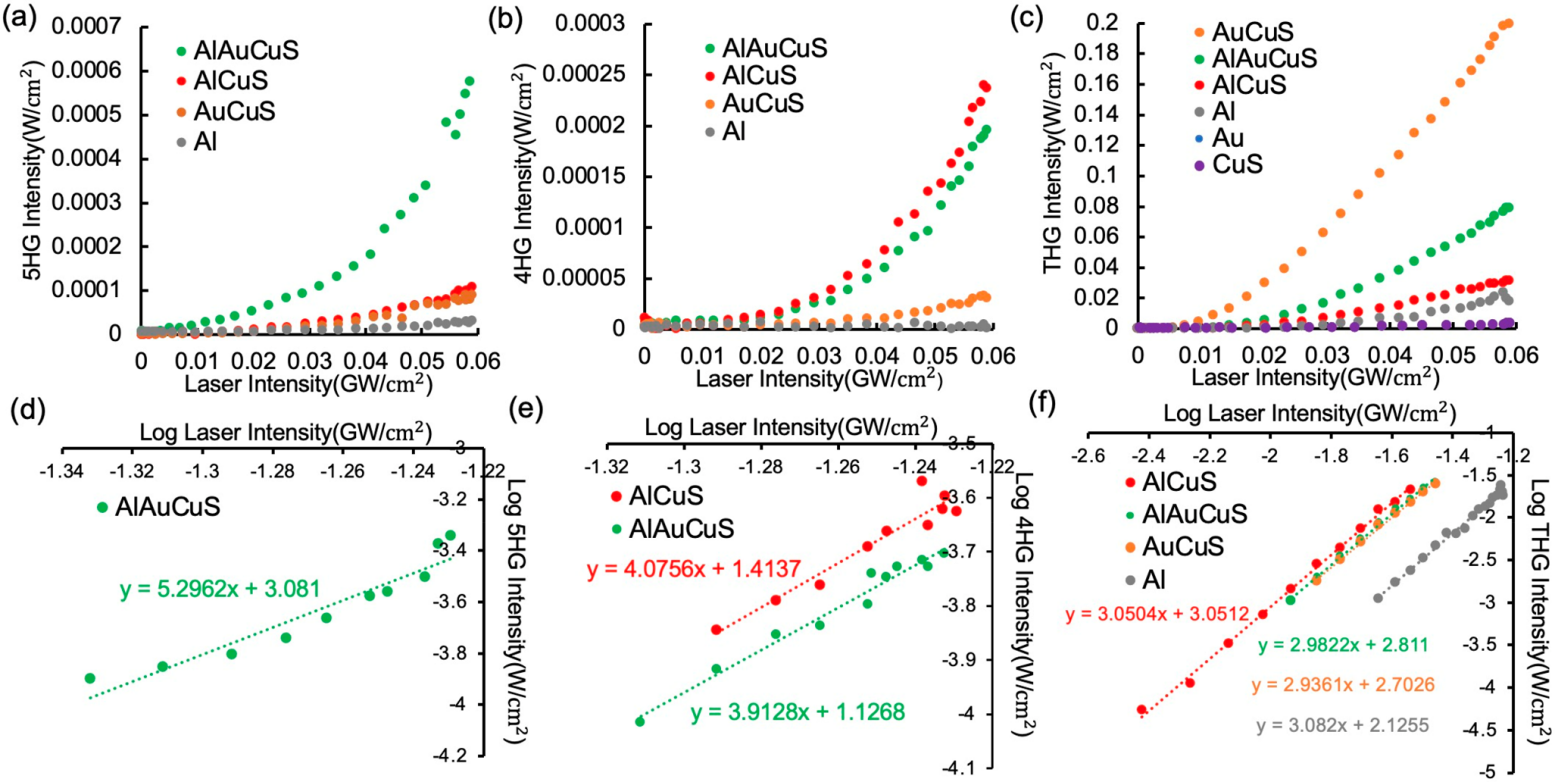
Intensities of (a) 5HG, (b) 4HG, and (c) THG signals as a function of input laser intensity for the nanoparticle films. Double-logarithmic plots of (d) 5HG intensity in AlAuCuS, (e) 4HG in AlAuCuS and AlCuS, and (f) THG in AlAuCuS, AlCuS, AuCuS, and Al as a function of pump laser intensity. Here, all of the heterostructure films contain 122 nm Al nanoparticles.

**Table 1:** Values of Nonlinearity Order and *R*^2^ of THG, 4HG, and 5HG Log–Log Fitting

fitting nonlinearity	THG	*R*^2^	4HG	*R*^2^	5HG	*R*^2^
Al/Au/CuS	2.98	0.99	3.91	0.97	5.29	0.91
Al/CuS	3.05	0.99	4.07	0.91	4.84	0.89
Au/CuS	2.93	0.99	3.62	0.98	4.47	0.96
Al	3.08	0.99	N/A	N/A	4.62	0.98

The 5HG intensity from Al/Au/CuS films is enhanced 6-fold compared to those of Au/CuS and Al/CuS films (Figure 2a) and 20-fold compared to the sum of 5HG from individual Al, Au, and CuS nanoparticle films (Figure S5a), strongly suggesting that surface plasmon resonances at the fundamental, second-harmonic, and third-harmonic frequencies in the triple-layer system enhance the upconverted process synergistically. This enhancement of 5HG can be attributed to two possible scenarios, as shown in panels a and b of Figure 3. The first scenario is based on a double sum-frequency (SF) cascade. Third harmonics are generated efficiently by plasmonic interactions between the Au and CuS in a THG cascade (SHG + SFG, ω + ω = 2ω, ω + 2ω = 3ω). Then, together with the THG created in aluminum LSPRs following three-photon absorption, third harmonics generated by Au NPs are transmitted to Al plasmons and combined with another second-harmonic photon, mostly generated in CuS, with a minor contribution from Au, to produce the final fifth harmonic emission in a second SFG process (3ω + 2ω = 5ω). The second scenario is a triple SF cascade, generating the fourth harmonics (3ω + ω = 4ω) and fifth harmonics (4ω + ω = 5ω) in an additional SF cascade. Both scenarios are possible, but the first (panel a) process is more probable for the observed enhancement, because highly efficient SF between 4ω and ω would require another plasmon resonance at 4ω.

**Figure 3:**
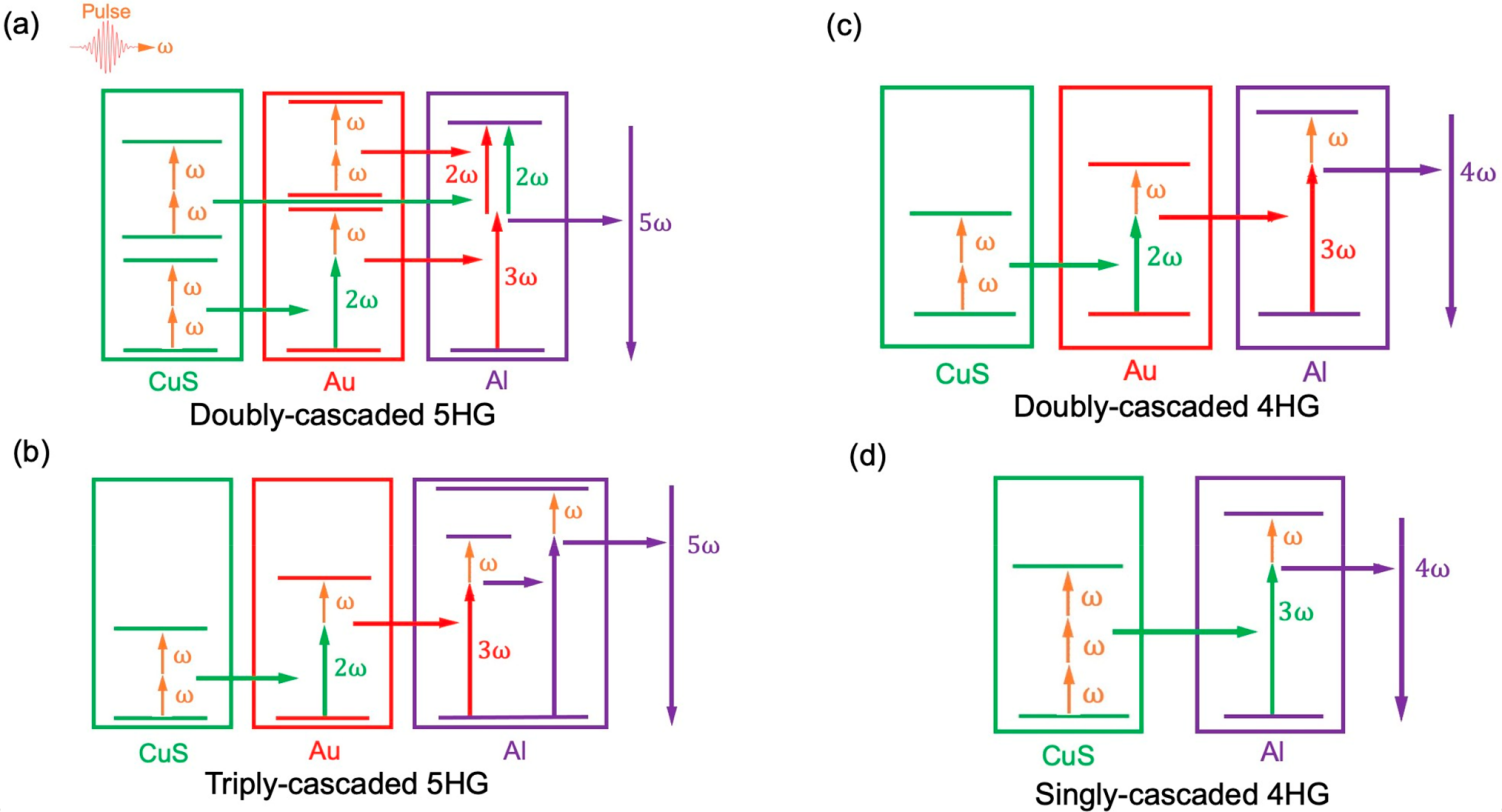
Schematic sum-frequency cascade mechanisms in plasmonic materials: (a) doubly cascaded 5HG in Al/Au/CuS films, (b) triply cascaded 5HG, (c) doubly cascaded 4HG in Al/Au/CuS films, and (d) singly cascaded 4HG in Al/CuS films.

The 4HG seems to be generated through multiple pathways that depend on specific nanocomposite structures. We demonstrate 7- and 165-fold enhancement for Al/Au/CuS films with respect to Au/CuS and single-component films, respectively (Figure 2b and Figure S5b), which we ascribe to the doubly cascaded 4HG mechanism (ω + 2ω = 3ω, 3ω + ω = 4ω) (Figure 3c). However, a similar 4HG intensity collected from Al/CuS films commands our attention. Without the 2ω resonances from Au NPs, there should be no effective cascaded THG; Figure S5b shows that a cascade of 2ω photons (2ω + 2ω = 4ω) from CuS is unlikely. Therefore, the enhanced 4HG is probably a singly cascaded process, in which third harmonics generated directly by THG in CuS are then transmitted to Al to interact with an incident pump photon to produce the fourth harmonic signal (ω + ω + ω = 3ω, 3ω + ω = 4ω), which is significantly enhanced by the plasmonic interactions between CuS and Al plasmons (Figure 3d). The fascinating observation of enhanced 4HG in the multiplasmonic structure suggests even-order-harmonic generation can be efficient in these composite structures, even when individual plasmonic materials in the heterostructure are centrosymmetric.

In summary, the Al/Au/CuS triple-layer system generates enhanced 5HG and 4HG by double SF cascades while Al/CuS hybrid films show significant 4HG enhancement by a singly cascaded mechanism. The harmonic conversion efficiencies (η_HG_) for these films are listed in Table 2, where η_*n*HG_ = *I*(*n*ω)/*I*(ω) (*n* = 3, 4, or 5). *I*(ω) is the maximum pump laser intensity we can achieve in experiments [*I*(ω) = 0.059 GW/cm^2^], while *I*(*n*ω) is the intensity of the *n*th harmonic signal collected at the maximum pump intensity seen in Figure 2. The enhanced THG in Al/Au/CuS and Au/CuS films is mediated by plasmon–plasmon coupling between CuS and Au NPs, while the relatively weaker THG output in Al/Au/CuS could be due to self-absorption at 3ω in Al NPs. The Al plasmon resonance produces a gigantic increase in 4HG conversion efficiency in Al/Au/CuS and Al/CuS films. The similar 4HG intensities from the Al/Au/CuS and Al/CuS films, which display distinct upconversion mechanisms, could be the result of multiple factors. On one hand, plasmonic interactions between CuS and Au in Al/Au/CuS could enhance cascaded third harmonics and intensify the 4HG efficiency compared to those of singly cascaded 4HG. On the other hand, in the triple-layer system, doubly cascaded 4HG and 5HG both require intermediate third harmonics in the final step; 4HG combines 3ω with the fundamental ω, while 5HG combines 3ω with 2ω. However, odd-order-harmonic generation (5HG) is more probable than even-order-harmonic generation (4HG) because of the centrosymmetry of the face-centered cubic structure of Al nanocrystals. Moreover, the much larger size of the Al NPs (122 nm) compared with those of Au (15 nm) and CuS (15 nm) NPs in these films decreases the surface:volume ratio and thus suppresses 4HG. The significant 5HG enhancement in Al/Au/CuS films indicates the critical importance of plasmonic interactions from plasmon resonances at fundamental, second harmonic, and third harmonic, by which both inputs (2ω and 3ω) seeded in the final SF process are resonantly enhanced, leading to the 5ω emission.

**Table 2:** Harmonic Generation Conversion Efficiencies (η_HG_) of the Plasmonic Heterostructure Films at Maximum Enhancement

	η_HG_ (×10^–9^)		
	THG	4HG	5HG
Al/Au/CuS	1.34	0.0033	0.0098
Al/CuS	0.53	0.0040	0.0018
Au/CuS	3.38	0.00051	0.0015
Al	0.31	0.00002	0.00042

It is also worth noting that in Al/Au/CuS and Au/CuS films the efficiencies of THG and 5HG are larger than the 4HG efficiency as a result of the symmetry requirement for even-order-harmonic generation. However, the opposite trend was observed in Al/CuS films, in which the efficiency of 4HG is significantly greater than that of 5HG. Without the second harmonic resonance, both SF processes involved in doubly cascaded 5HG (ω + 2ω = 3ω, 3ω + 2ω = 5ω) are deeply suppressed due to insufficient seeding of second harmonics, resulting overall in much less 5HG. Additionally, measurements of Al/Au/CuS with surface plasmon resonances of Al NPs blue- and red-shifted from 350 nm (Figure S6) show that efficient 5HG and 4HG enhancements largely disappear, strongly indicating the indispensable contribution of aluminum LSPRs to the generation of these harmonics.

To better understand the resonant enhancement effect on high-order-harmonic generation, we simulate the electric-field distribution at the fifth- and fourth-harmonic frequencies under the fundamental pump in the Al/Au/CuS heterostructure using the finite-difference time-domain (FDTD) calculation (for details, see Method in the Supporting Information). The simulated absorption spectrum is quite consistent with the experimental profile, indicating three distinct LSPRs (Figure S8). The simulated *E*_fundamental_ demonstrates the significant localized-field enhancement arising from plasmon–plasmon coupling (Figure 4a). Moreover, the strongest *E*_5HG_ and *E*_4HG_ occur between the Au and Al nanoparticle, in accordance with the cascaded mechanism that suggests the final SF step, in which the fifth and fourth harmonics are generated, should be greatly enhanced by the coupling between Au and Al plasmons (Figure 4b,c).

**Figure 4:**
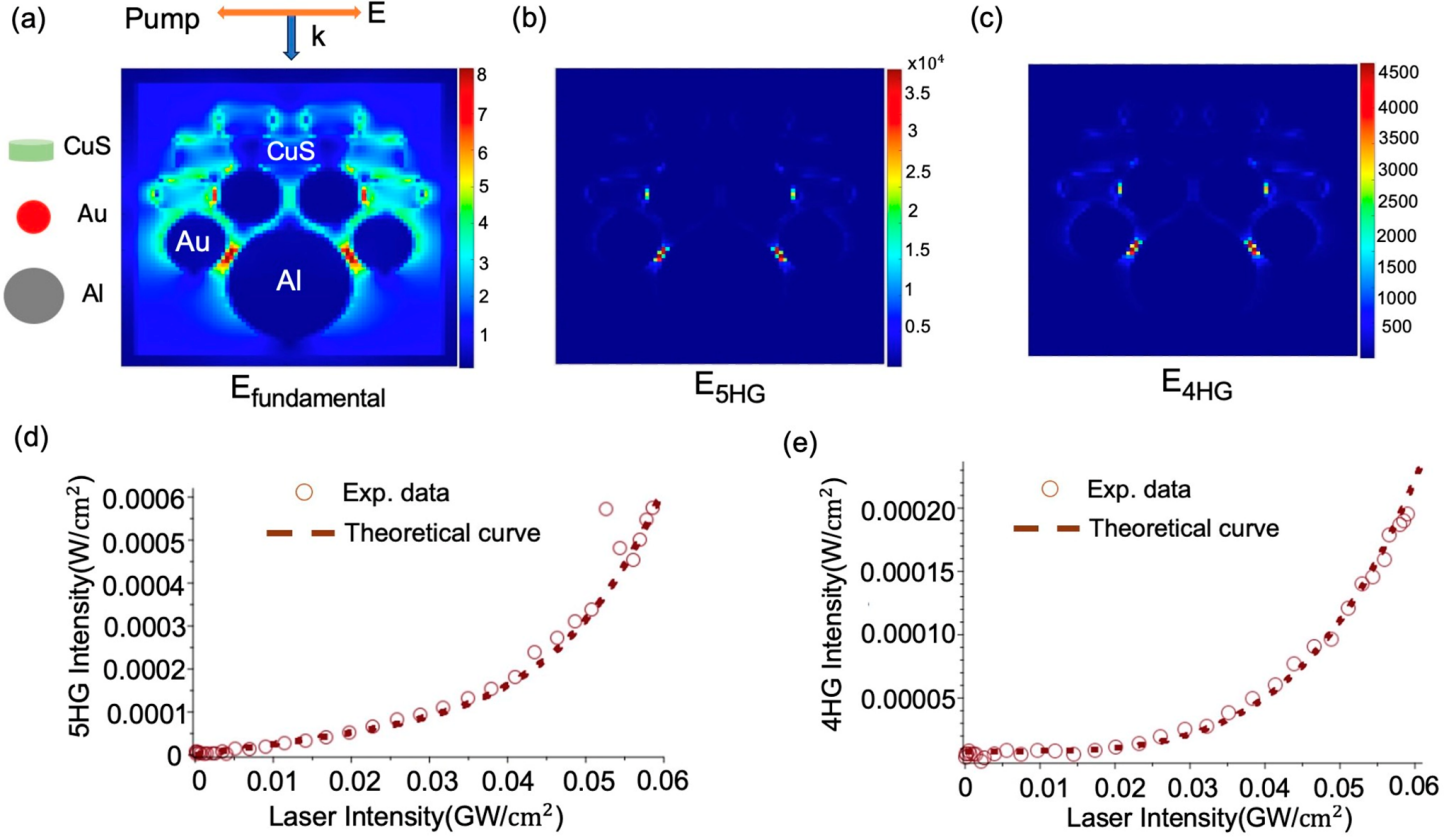
Simulated electric-field distribution at the (a) fundamental, (b) fifth-harmonic, and (c) fourth-harmonic frequency. The color scale bars show the relative increase in field enhancement. Correlation between experimental results and predicted values from the dipole–dipole analytical model for the Al/Au/CuS films (122 nm Al). (d) 5HG and (e) 4HG signals as a function of input intensity predicted from the analytical model for Al/Au/CuS heterostructure films are presented as dotted lines. The experimental data (Figure 2a,b) are plotted as empty circles.

To further support this hypothesis, we developed an analytical model for the 5HG and 4HG intensities of Al/Au/CuS nanohybrids. In addition to the pump field, we consider the electric field incident on NPs from the surface plasmon polariton (SPP) and the dipole–dipole interaction (DDI). Pump, SPP, and DDI fields all participate in generating 5HG and 4HG. However, the interaction between the coupled fields is incoherent because the plasmon dephasing time is much shorter than the pump-pulse duration, meaning that LSPRs lose phase coherence and thermalize by electron–electron scattering even before each pump pulse ends.

In our model, the 5HG and 4HG output intensities are the sum of the harmonic emission from each nanoparticle component. Because the calculations for each harmonic are similar, we take as an example the 4HG produced by Al/Au/CuS films; more details of the model are included in the Supporting Information. Here, we describe the formalism briefly and exhibit the final expressions.

There are four electric fields incident on Al NPs: the pump field, SPP fields from Au and CuS NPs, and the total DDI electric field. The SPP fields are generated by the interaction between the incident photons and the surface plasmons. They have the form^42−44^
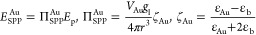
1
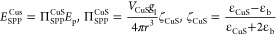
2where Π_SPP_^Au^ and Π_SPP_^CuS^ are SPP coupling constants. ζ_Au_ and ζ_CuS_ are SPP polarization factors, which become extremely large when the pump field excites the LSPR of NPs, because the denominator vanishes. The volume factors of Au and CuS NPs are *V*_Au_ and *V*_CuS_, respectively, while ε_Au_, ε_CuS_, and ε_b_ are the dielectric constants of the Au, CuS, and the substrate, respectively. *g*_l_ is the polarization parameter. The total DDI field originates from interactions among the ensemble of homogeneous dipoles and falls on three NPs as follows:
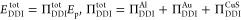
3
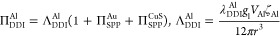
4
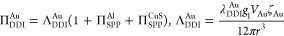
5
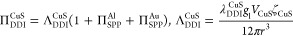
6Total DDI coupling parameter Π_DDI_^tot^ is the sum of the single DDI coupling parameter Π_DDI_^Al^, Π_DDI_^Au^, and Π_DDI_^CuS^, where Λ_DDI_^Al^, Λ_DDI_^Au^, and Λ_DDI_^CuS^ are the DDI parameters and λ_DDI_^Al^, λ_DDI_^Au^, and λ_DDI_^CuS^ are the DDI constants.

After building expressions for all electric fields incident on aluminum NPs, we can calculate the amplitude of the 4HG field from coupled three-wave theory as
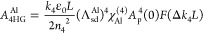
7where
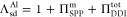
8
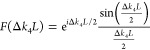
9

For the sake of simplicity, we consider only the phase matching condition, so the phase function is unity. The two SPP fields from Au and CuS NPs are combined into one total SPP field denoted as *E*_SPP_^m^. *k*_4_ and *n*_4_ are the wavevector and refractive index, respectively, evaluated for 4HG, and *A*_p_ is the amplitude of the pump field. The fourth-order susceptibility is obtained using the density matrix method as follows:
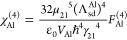
10where *F*_Al_^(4)^ is a dimensionless quantity. The 4HG intensity can be described as
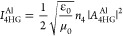
11

Inserting eqs 7 and 10 into eq 11, we obtain the final statement:
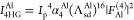
12where
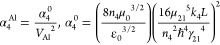
13

Similarly, following the method of Al NPs, we can obtain the 5HG and 4HG intensities for Au and CuS NPs. Thus, the general expressions for 4HG and 5HG from the Al/Au/CuS triple-layer system are
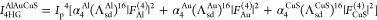
14
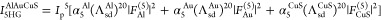
15

The upconversion properties of these films are accurately predicted by the model. As shown in panels d and e of Figure 4, the enhanced fifth and fourth harmonic signals in Al/Au/CuS are well-reproduced by plotting eqs 14 and 15 using SPP and DDI coupling constants Π_SPP_^m^ and Π_DDI_^m^, respectively, as the fitting parameters by the linear least-squares fitting method, where Π_SPP_^m^ = Π_DDI_^m^ = 2.64.

Moreover, the model shows that the enhanced 4HG/5HG signals in these heterostructure films can be attributed to plasmon–plasmon coupling by combining additional SPP and DDI fields to enhance the harmonic emission. Equations 1–6 demonstrate that the SPP and DDI fields are proportional to the polarization factor, which becomes significant only when the pump frequency is near the plasmon resonance. As we propose, the 5HG and 4HG in the Al/Au/CuS films are both doubly cascaded, during which CuS, Au, and Al plasmons are excited by the fundamental intermediate second- and third-harmonic photons, respectively. In this way, the additional coupling terms including the SPP and DDI field from Au and Al NPs are non-zero even under the 1050 nm excitation and account for the huge increase in the 5HG and 4HG intensities.

In summary, we fabricated a triple-layer plasmonic heterostructure containing CuS, Au, and Al NPs, which exhibited LSPRs at the fundamental, second-harmonic, and third-harmonic frequencies of the 1050 nm pump photons. The 4HG and 5HG intensities demonstrate 7- and 6-fold enhancement, respectively, in Al/Au/CuS compared to Au/CuS hybrid films. The crucial effect of aluminum plasmon resonances at 350 nm on the upconversion mechanisms is indicated by the observation of attenuated 5HG when shifted from the third-harmonic resonance by synthesizing Al NPs of varying sizes. Additionally, we propose that upconversion in these films results from a cascade of SF steps mediated by surface plasmon resonances. While the 4HG in Al/CuS films is a singly cascaded THG plus SFG process, the 5HG and 4HG in Al/Au/CuS films both involve a doubly cascaded mechanism; that is, the final generated fifth or fourth harmonics are produced in the SFG between a third harmonic photon (produced in turn by cascaded THG) and a second harmonic or fundamental photon, respectively. An analytical model based on the dipole–dipole interaction not only reproduces the experimentally observed enhancements but also supports our cascaded theory when the SPP and DDI fields from each nanoparticle component are considered. These observations of 4HG and 5HG in Al/Au/CuS films provide an important new synthetic route for creating harmonically resonant plasmonic heterostructures that show an efficient enhancement of high-order-harmonic generation.

## Supporting Information Available

Method, including simulation, nanoparticle synthesis, and structural and optical characterization; 5HG, 4HG, and the log–log plot for Al/Au/CuS (containing 98 and 147 nm Al NPs); simulated absorption spectrum; and theoretical dipole–dipole interaction model of 4HG and 5HG from Al/Au/CuS heterostructure films (PDF)
