# Supplementary material for: Harmonic Generation up to Fifth Order from Al/Au/CuS Nanoparticle Films
Source: Nano Lett. 2024 Apr 15;24(16):5085–92. doi: 10.1021/acs.nanolett.4c00776 (PMC11057033; doi:10.1021/acs.nanolett.4c00776)
Supplement: Supplementary file 1 — nl4c00776_si_001.pdf [file nl4c00776_si_001.pdf]

Supplementary Information

Harmonic Generation up to Fifth Order from Al/Au/CuS Nanoparticle Films

*Yueming Yan, Nathan J. Spear, Qingzhou Meng, Mahi R. Singh, Janet E. Macdonald, Richard F. Haglund**

Y. Yan, R. F. Haglund
Department of Physics and Astronomy
Vanderbilt University
Nashville, Tennessee 37235 United States of America
E-mail: richard.haglund@vanderbilt.edu

N. J. Spear
Interdisciplinary Materials Science
Vanderbilt University
Nashville, Tennessee 37235, United States of America

J. E. Macdonald
Department of Chemistry
Vanderbilt Institute of Nanoscale Science and Engineering
Vanderbilt University
Nashville, Tennessee 37235 United States of America

Q. Meng, M. R. Singh
Department of Physics and Astronomy
The University of Western Ontario
London N6A 3K7, Canada

Section S1. Method

Here we describe the optical simulation, nanoparticle synthesis for CuS, Au and Al and the structural and optical characterization with X-ray diffraction, transmission electron microscope, profilometry, UV-vis-NIR spectrophotometer. The harmonic generation measurements were conducted with self-constructed nonlinear setup.

Simulation

The theoretical simulations of the absorption spectra and near-field distributions were executed on commercially available software (Ansys Lumerical FDTD 2021 R2) using a finite-difference time-domain (FDTD) solver. The material properties of the Au nanosphere (15nm diameter) were taken from the Johnson and Christy dataset[1], while the Al sphere (30nm diameter) and CuS polygon (base side length 7.3nm, 6.7nm height) were modeled as a Drude plasma. The damping constants and plasma frequency of these models were adopted from the reference [2,3]. In our simulated structure, the large Al nanosphere is surrounding by several Au and CuS nanoparticles. The plane wave centered at 1050nm was used as the incident pump. The grid size in all axes was set to 1nm and periodic boundary conditions were employed. The linear near-field distribution at the fundamental frequency was collected and used as a source to obtain the nonlinear electric field distribution at the fourth and fifth harmonic frequency.

Nanoparticle synthesis

Nanoparticles of CuS and Au were synthesized using solvothermal techniques as described in previous reports[3,4], with some adjustments. Aluminum nanoparticles were synthesized in the reaction:

$$(CH_3)_2C_2H_5NAlH_3 \xrightarrow[(2)\,\frac{\text{Oleic Acid}}{\text{1,4-dioxane}}]{(1)\,\frac{Ti(OPr)_4}{\text{Toluene/THF}}} Al + H_2 + (CH_3)_2C_2H_5N$$

All reagents were procured from Sigma-Aldrich in Sure-Seal™ bottles. To make NPs with a broad plasmon resonance in the UV, 10 mL of dry toluene was combined with 5 mL of dry tetrahydrofuran under argon and stirred at room temperature for 1 hour. Then 3 mL of 0.5 M (in toluene) N,N-dimethylethylamine alane was injected to the solvent mixture followed immediately by 0.025 mL of neat titanium (IV) isopropoxide. Unlike Al nanoparticle syntheses in literature[5], injecting neat titanium isopropoxide rather than a solution in toluene eased the air-free requirements on the reagent preparation—which will form TiO_2 if exposed to air or water—allowing the synthesis to be performed on a Schlenk line without first preparing reagents in a glovebox. The reaction mixture quickly turned from clear to dark brown upon injection of the titanium catalyst. This reaction was allowed to continue at room temperature for 24 hours during which time the mixture turned gray. To terminate the reaction, 0.25 mL of 250 mM oleic acid in dioxane was added to the product. Cleanup of the product NPs proceeded by the addition of 30 mL of isopropanol and centrifugation at 8000 rpm for 5 minutes. The reaction byproducts were then decanted from the precipitated NPs and the nanoparticle product was resuspended in toluene: this procedure was repeated twice to ensure complete removal of byproducts and unreacted aluminum. Notably, reaction byproducts contain dimethylethylamine, which possesses a strong piscine odor. A 5% (by volume) acetic acid solution was used to clean the glassware after the product was collected to solubilize the amine byproduct thereby limiting the impact of the stench on unsuspecting coworkers.

Structural characterization

Powder X-ray diffraction (XRD) patterns of the NPs were acquired in a Rigaku SmartLab X-ray diffractometer with a CuKα source and a D/teX Ultra 250 detector: operating voltage was 40 kV and current was 44 mA. The powder X-ray diffraction patterns of synthesized 122nm Al nanoparticles are shown in Figure S1. Comparing with JCPDS data, the results are in good agreement with the face-centered cubic form of aluminum, whose most characteristic diffraction peaks are highlighted. The size and geometry of the Al NPs were measured in a Tecnai Osiris transmission electron microscope equipped with a quad Super-X detector operated at 200keV. The thickness of the heterostructure films was measured by the Bruker Dektak 150 profilometer.

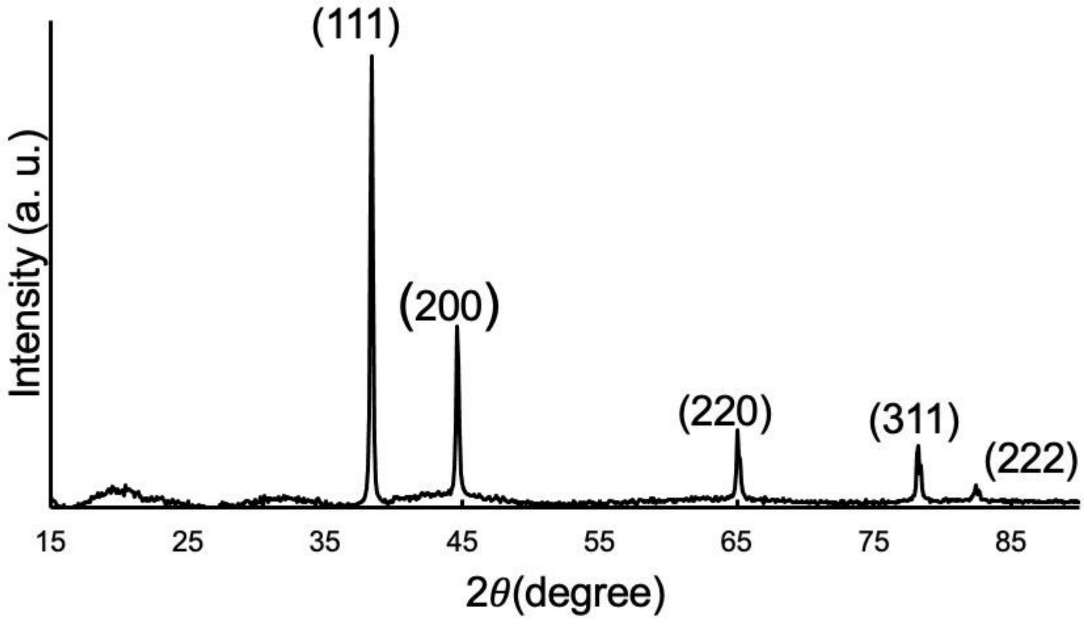

Figure S1. X-ray diffraction (XRD) patterns of the 122nm aluminum nanoparticles.

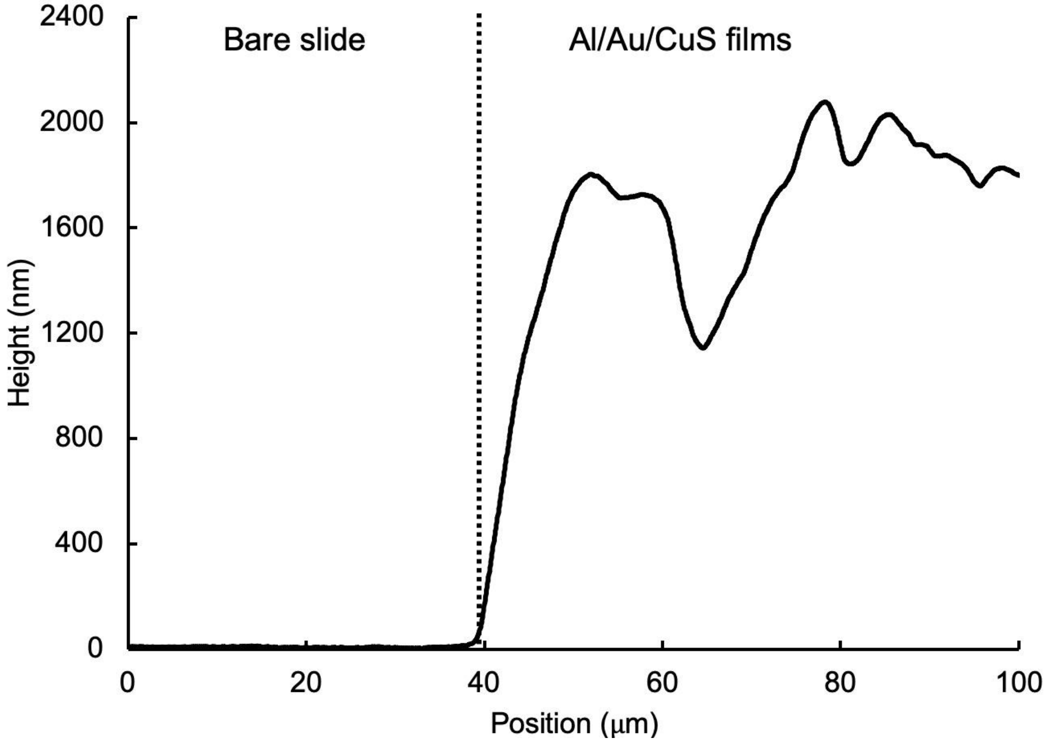

Figure S2. Profilometry measurement of film thickness. Low positions (before dotted line) correspond to bare substrate slide where the nanoparticle films have been wiped away. Difference in average height between bare substrate and film domains (film height) is 1734 nm.

Optical measurements

Extinction spectra of nanoparticle films were acquired in a UV-vis-NIR spectrophotometer with an integrating sphere from 300 to 1400nm (Jasco V-670). In Figure S3, we show the absorption spectrum of 98nm, 122nm and 147nm aluminum nanoparticles. Only 122nm Al nanoparticles exhibits the LSPR at 350nm. As the diameter increases to 147nm, the plasmon resonance red-shifts to 375nm; as the diameter decreases to 98nm, the resonance peak blue-shifts to 280nm. The position of the LSPR depends on the size of aluminum nanoparticles. Harmonic generation from the films was measured in the nonlinear optical microscope depicted in Figure S4. The Orpheus-F optical parametric amplifier (OPA) was pumped by a compact, high repetition-rate femtosecond Pharos laser comprising an oscillator and chirped-pulse amplifier, employing diode-pumped Yb:KGW as an active medium and generating pulses centered at 1035$\pm$5nm at an average power of 600 mW, 187 fs duration, at a repetition rate of 60 kHz and an energy per pulse of 10 μJ. The signal and idler pulses of the OPA are tunable over 650-1000nm and 1000-2500nm ranges, respectively, providing gap-free tunability. Here, the idler beam tuned to 1050nm was picked as the pump beam and mechanically chopped at a frequency of 265 Hz, with a duty cycle of 20%. This avoided repetitive exposures and ensured stable measurement conditions. To determine the dependence of upconverted signal on the pump intensity, we used a rotational polarizer to adjust the laser power by varying the angle between the fixed output pump polarization of the pump laser and the axis of the rotational polarizer. Pump power was measured in a Thorlabs S130C power meter with a PM100D readout.

The 4HG and 5HG signals were isolated from the other upconverted light signals using appropriate band pass filters (Optosigma VPF-25C-10-12-26500 for 4HG and VPF-25C-10-12-21400 for 5HG). The detector was the solid-state photomultiplier tube (PMT, Hamamatsu, R9875U for ultraviolet light detection) operating at 1.1 kV.

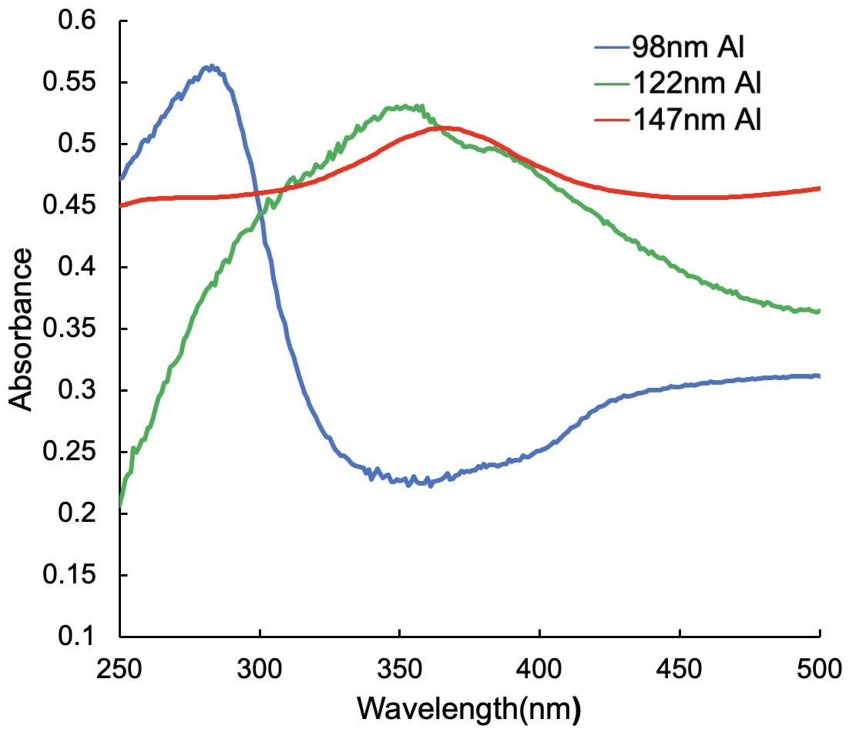

Figure S3. UV-vis-NIR spectrum of AlAuCuS films containing the aluminum nanoparticles of the average diameter 98nm (blue), 122nm (green), 147nm (red).

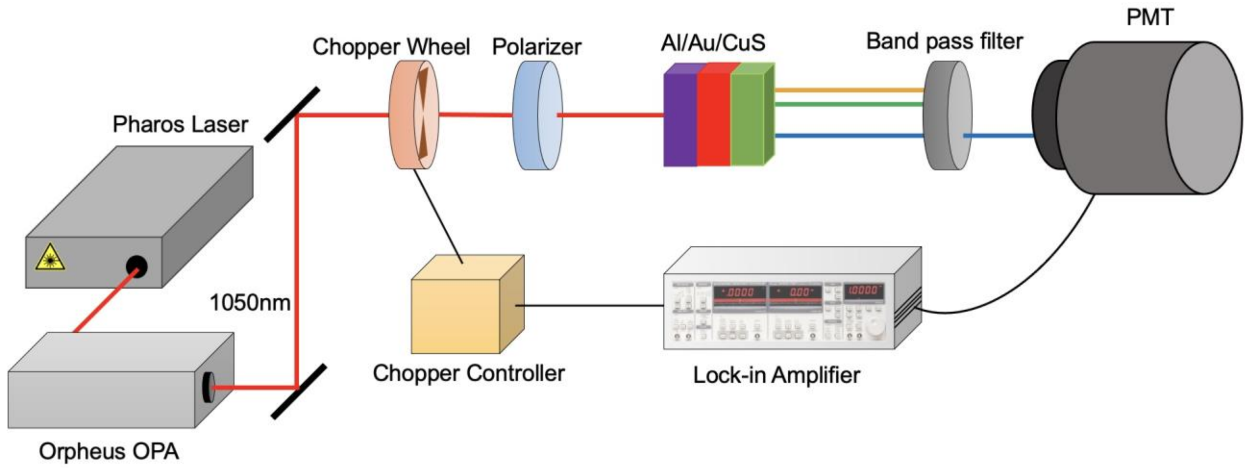

Figure S4. Experimental configuration for measuring harmonic generation from Al/Au/CuS triple-layer films.

Section S2. Supplementary Figure

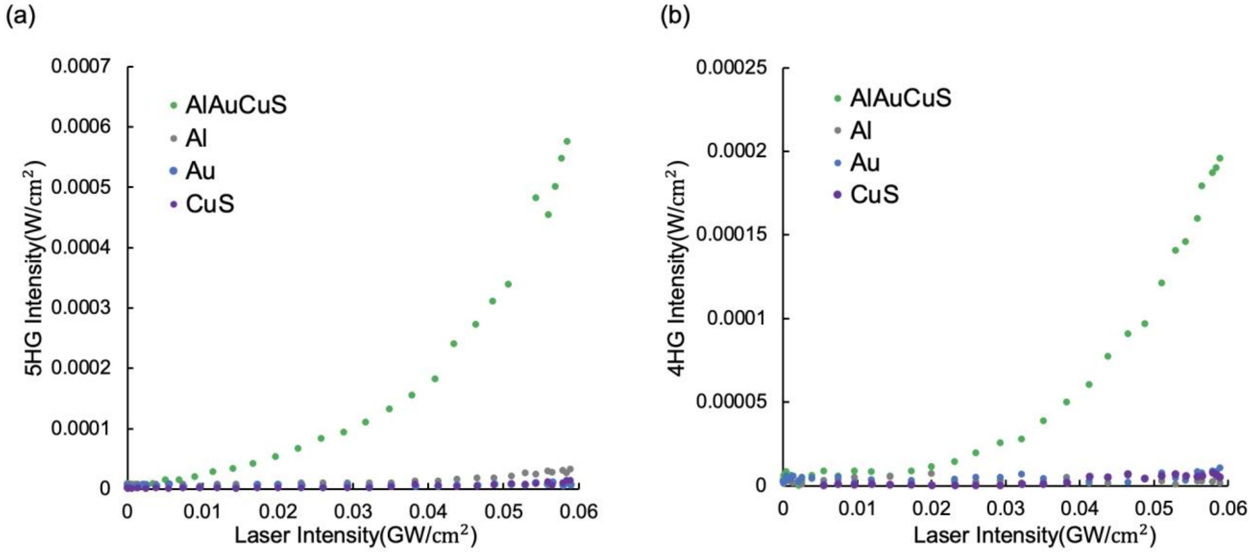

Figure S5. Intensity of (a) 5HG and (b) 4HG signals as a function of the input laser intensity for the AlAuCuS (122nm Al), Al (122nm), Au and CuS films.

In Figure S5, we compare 5HG and 4HG intensity from Al/Au/CuS films with single component films: Al, Au CuS only. We demonstrate twenty-fold enhanced 5HG and 165-fold enhanced 4HG in the triple-layer system with respect to the constituent films due to the harmonically plasmonic interactions.

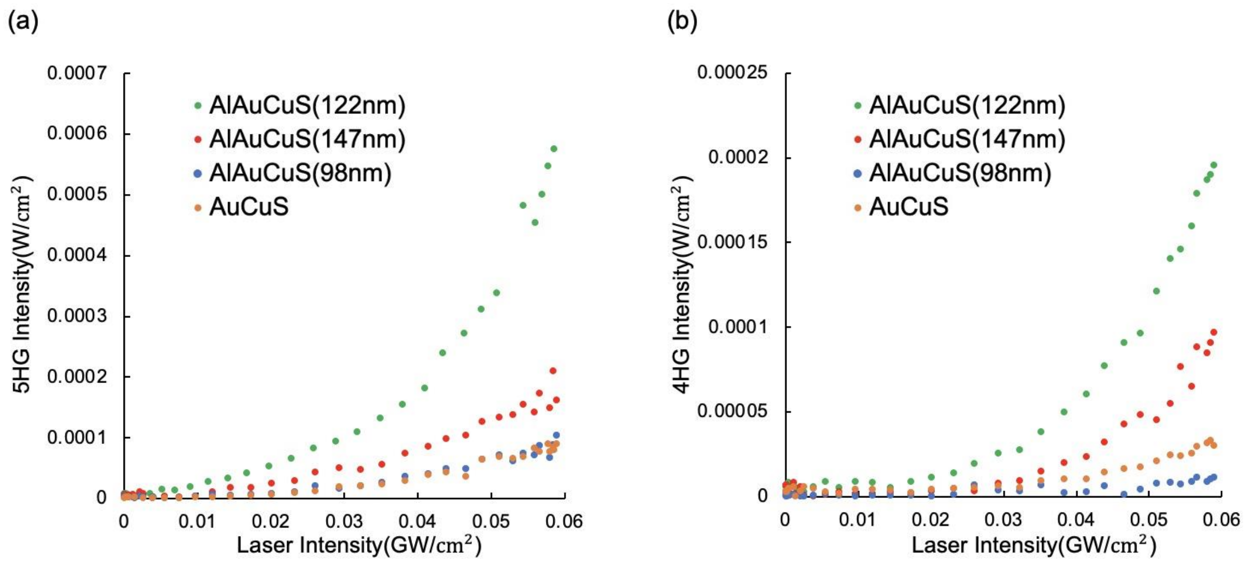

Figure S6. Intensity of (a) 5HG and (b) 4HG signals as a function of input laser intensity for the AlAuCuS films with different aluminum nanoparticle diameters and AuCuS hybrid films.

In Figure S6, we compare the 5HG and 4HG intensities from Al/Au/CuS films containing Al nanoparticles with different diameter. We observe that when the LSPRs of Al nanoparticles are shifted from the third harmonic frequency (350nm), the enhancement effect of the harmonic generation is significantly attenuated, indicating the critical importance of the harmonic condition between coupled nanoparticle LSPRs for efficient plasmonic interactions.

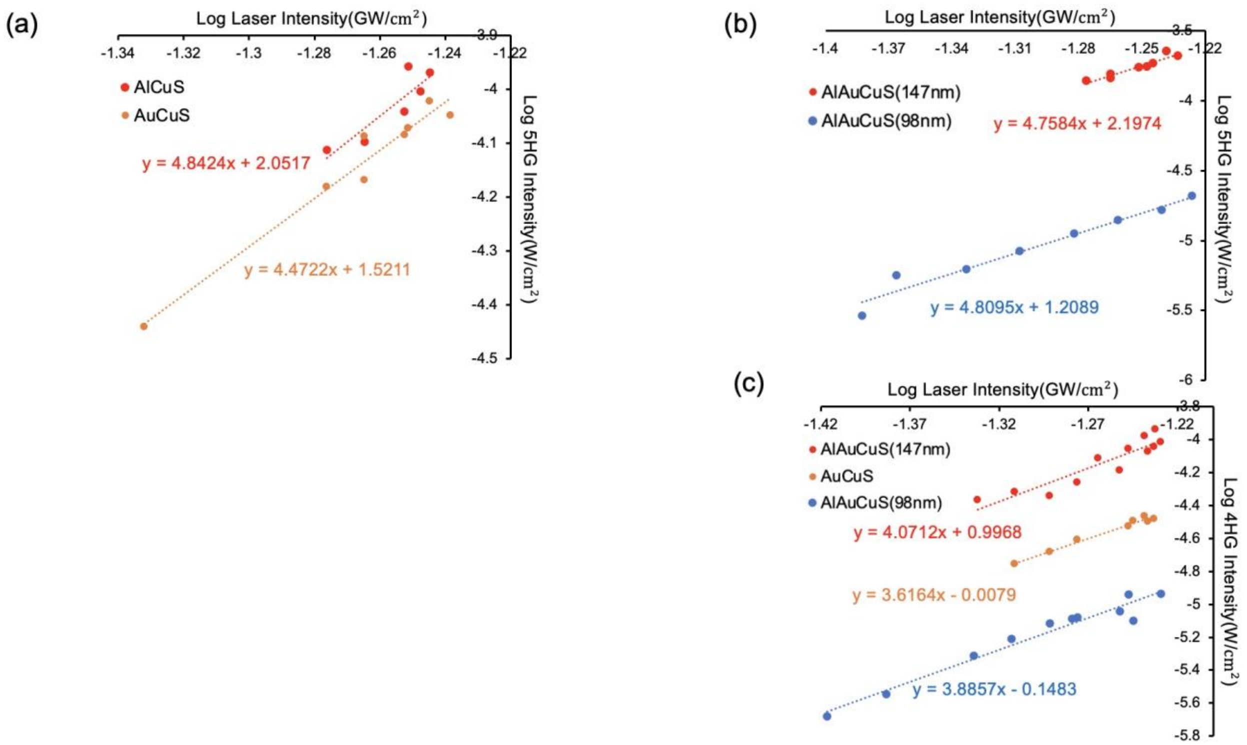

Figure S7. Double-logarithmic plots of (a) 5HG intensity in AlCuS (122nm Al) and AuCuS (b) 5HG intensity in AlAuCuS (147nm Al) and AlAuCuS (98nm Al) (c) 4HG intensity in AlAuCuS (147nm Al), AlAuCuS (98nm Al) and AuCuS.

In Figure S7, we show the log-log plots of 5HG and 4HG intensity as a function of pump laser intensity in Au/CuS, AlCuS and Al/Au/CuS films containing 98nm Al and 147nm Al nanoparticles, respectively. The linear fitting of the plots gives the nonlinear order of the detected harmonic generation.

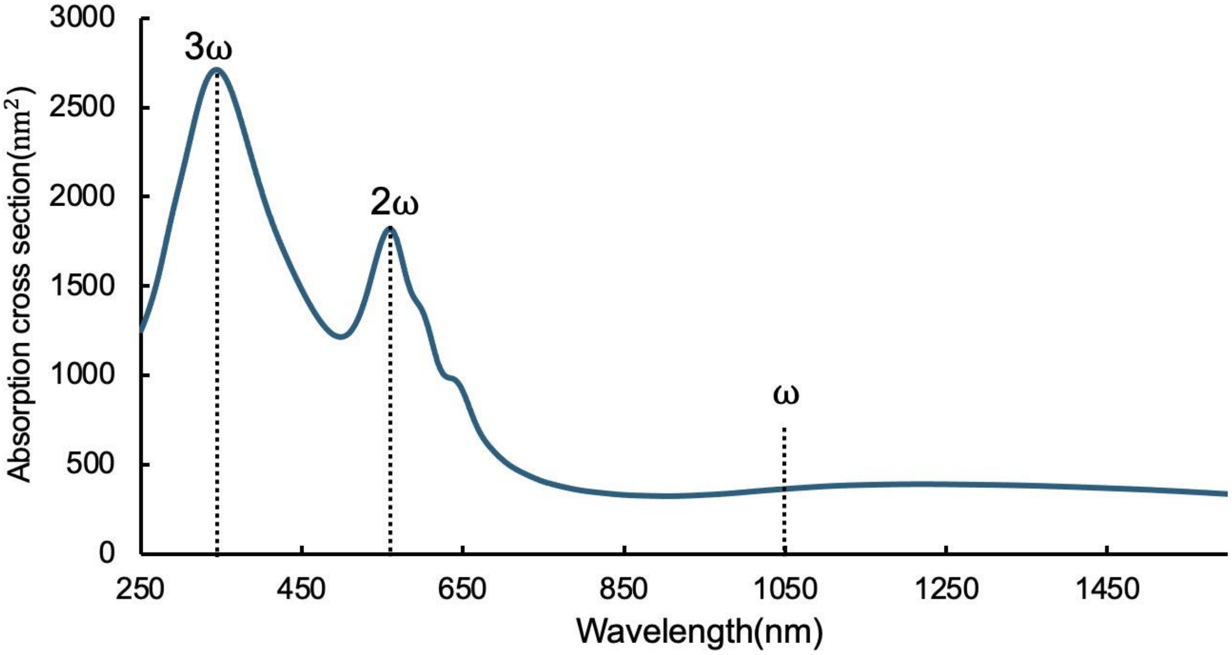

Figure S8. Simulated absorption spectrum of the AlAuCuS heterostructure system.

In Figure S8, the simulated absorption spectrum from FDTD simulation successfully reproduced three distinct LSPR peaks at the fundamental (1050nm), second harmonic (525nm) and third harmonic (350nm) of the 1050nm pump frequency, respectively.

Section S3. A Theory of Fourth Harmonic Generation and Fifth Harmonic Generation in Al/Au/CuS Heterostructure films

Here, a theory of fourth harmonic generations (4HG) and fifth harmonic generations (5HG) is developed for plasmonic heterostructures made of an ensemble of Au, Al and CuS nanoparticles (NPs). Coupled-mode formalism based on Maxwell's equations is used to obtain the intensity of the output 4HG and 5HG light. It is found that the phenomena of 4HG and 5HG depend on the fourth and fifth-order susceptibilities, which are evaluated by the density matrix method. Analytical expressions for the intensities of the 4HG and 5HG are calculated in the presence of the surface plasmon polaritons (SPPs) assuming a dipole-dipole interaction (DDI). We have predicted the enhancement of the 4HG and 5HG due to the SPP and DDI coupling.

3.1 SPP and DDI fields in plasmonic nanoparticles

We consider that a nanohybrid comprising three layers of plasmonic nanoparticles (PNPs). The top, middle and bottom layer contain an ensemble of CuS NPs, Au NPs and Al NPs, respectively. They are deposited on a silicon substrate (*i.e.*, background material). A schematic diagram of the nanohybrid structure is shown in Figure S9.

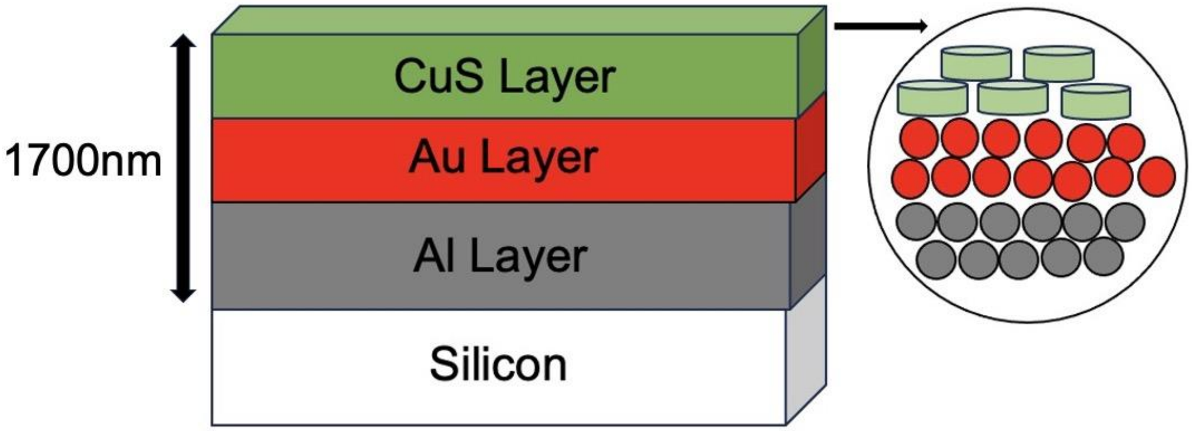

Figure S9. Schematic diagram of the plasmonic films which consist of three layers of the nanohybrids.

Now we calculate the SPP and DDI fields produced by the ensemble of Au, Al and CuS NPs.

3.1.1 Surface Plasmon Polariton Field

Free electrons on the surface of the metallic nanoparticles (MNPs) oscillate collectively and produce electron surface plasmons. However, CuS is a direct-gap, semiconductor. In the present work, CuS NPs are heavily doped p-type semiconductors. The p-type semiconductors have positively charged holes which are free to move in materials. Hence, the CuS NPs have free holes on the surface and these holes oscillate collectively to produce hole surface plasmons.

When a probe field is applied on the PNPs, photons of the probe field interact with the surface plasmons to create surface plasmon polaritons (SPPs). Therefore, the Au, Al and CuS NPs create their own SPP fields. Then we calculate the SPP electric fields produced inside the triple-layer system. The dielectric constants of the Au NPs, Al NPs and CuS NPs are denoted as ϵ_{Au}, ϵ_{Al} and ϵ_{CuS}, respectively. The dielectric constant of the silicon background is ϵ_b. The volumes of three nanoparticles are denoted as V_{Au}, V_{Al} and V_{CuS}. We apply a monochromatic probe field with amplitude E_p and frequency ω_p in the nanohybrid. The probe field induces the dipoles in the Au NPs, Al NPs, and CuS NPs, and they are denoted as P_{SPP}^{Au}, P_{SPP}^{Al} and P_{SPP}^{CuS}. These dipoles produce the SPP fields denoted as E_{SPP}^{Au}, E_{SPP}^{Al}, and E_{SPP}^{CuS}. They are called Au-SPP, Al-SPP and CuS-SPP fields, respectively. Solving the Maxwell's equations in the quasi-static approximation[6-8], we can obtain the following expressions of SPP fields

$$E_{SPP}^{Au} = \frac{P_{SPP}^{Au}}{4\pi\epsilon_0\epsilon_b r^3}$$

$$E_{SPP}^{Al} = \frac{P_{SPP}^{Al}}{4\pi\epsilon_0\epsilon_b r^3}$$

$$E_{SPP}^{CuS} = \frac{P_{SPP}^{CuS}}{4\pi\epsilon_0\epsilon_b r^3} \quad (1)$$

where

$$P_{SPP}^{Au} = \epsilon_0\epsilon_b V_{Au} g_l \zeta_{Au}(E_p)$$

$$P_{SPP}^{Al} = \epsilon_0\epsilon_b V_{Al} g_l \zeta_{Al}(E_p)$$

$$P_{SPP}^{CuS} = \epsilon_0 \epsilon_b V_{CuS} g_l \zeta_{CuS}(E_p) \quad (2)$$

Here the ζ_{Au}, ζ_{Al} and ζ_{CuS} quantities are called the SPP polarization factors and found as follows

$$\zeta_{Au} = \left[\frac{\epsilon_{Au} - \epsilon_b}{\epsilon_{Au} + 2\,\epsilon_b}\right]$$

$$\zeta_{Al} = \left[\frac{\epsilon_{Al} - \epsilon_b}{\epsilon_{Al} + 2\,\epsilon_b}\right]$$

$$\zeta_{CuS} = \left[\frac{\epsilon_{CuS} - \epsilon_b}{\epsilon_{CuS} + 2\epsilon_b}\right] \quad (3)$$

In the eqns. (2), the constant g_l is called the polarization parameter and it has values $g_l = 1$ and $g_l = -2$ for $P_{SPP}||E_{SPP}$ and $P_{SPP} \perp E_{SPP}$, respectively. Inserting eqn. (2) into eqn. (1), we get the SPP fields as follows

$$E_{SPP}^{Au} = \Pi_{SPP}^{Au} E_p$$

$$E_{SPP}^{Al} = \Pi_{SPP}^{Al} E_p$$

$$E_{SPP}^{CuS} = \Pi_{SPP}^{CuS} E_p \quad (4)$$

where

$$\Pi_{SPP}^{Au} = \frac{V_{Au} g_l}{4\pi r^3} \zeta_{Au}$$

$$\Pi_{SPP}^{Al} = \frac{V_{Al} g_l}{4\pi r^3} \zeta_{Al}$$

$$\Pi_{SPP}^{CuS} = \frac{V_{CuS} g_l}{4\pi r^3} \zeta_{CuS} \quad (5)$$

Note that all three electric fields depend on r^{-3}. The Π_{SPP}^{Au}, Π_{SPP}^{Al}, and Π_{SPP}^{CuS} parameters are unitless SPP coupling constants.

3.1.2 Dipole-Dipole Interaction Field

Now, we calculate the dipole-dipole interaction in the ensemble of PNPs. When the incident probe field falls on the PNPs, it induces dipoles that interact with each other via the dipole-dipole interaction. Let us first calculated the DDI field produced by the ensemble of Au NPs. There are three electric fields incident on the Au NPs: the probe field, the Al-SPP field and CuS-SPP field. All three fields induce dipoles in the ensemble of Au NPs that interact with each other via the DDI and produce a DDI field. Following the method in the literature [6], the DDI field is found as follows

$$E_{DDI}^{Au} = \Lambda_{DDI}^{Au}\left(E_p + E_{SPP}^{Al} + \Pi_{SPP}^{CuS}\right)$$

$$\Lambda_{DDI}^{Au} = \frac{\lambda_{DDI}^{Au} g_l V_{Au} \zeta_{Au}}{12\pi r^3} \quad (6)$$

where Λ_{DDI}^{Au} is called the Au DDI parameter. Inserting the expressions of the SPP electric fields from eqn. (4) into eqn. (6), we get

$$E_{DDI}^{Au} = \Pi_{DDI}^{Au} E_p$$
$$\Pi_{DDI}^{Au} = \Lambda_{DDI}^{Au}\left(1 + \Pi_{SPP}^{Al} + \Pi_{SPP}^{CuS}\right) \quad (7)$$

where Π_{DDI}^{Au} is the Au DDI coupling parameter. Similarly, the probe field also induces the dipoles in Al NPs and CuS NPs that interact with each other via the DDI. Following the method in Au NPs, we obtain the DDI fields produced by Al NPs and CuS NPs as follows

$$E_{DDI}^{Al} = \Pi_{DDI}^{Al} E_p$$
$$\Pi_{DDI}^{Al} = \Lambda_{DDI}^{Al}\left(1 + \Pi_{SPP}^{Au} + \Pi_{SPP}^{CuS}\right)$$
$$\Lambda_{DDI}^{Al} = \frac{\lambda_{DDI}^{Al} g_l V_{Al} \zeta_{Al}}{12\pi r^3} \quad (8)$$

$$E_{DDI}^{CuS} = \Pi_{DDI}^{CuS} E_p$$
$$\Pi_{DDI}^{CuS} = \Lambda_{DDI}^{CuS}\left(1 + \Pi_{SPP}^{Au} + \Pi_{SPP}^{Al}\right)$$
$$\Lambda_{DDI}^{CuS} = \frac{\lambda_{DDI}^{CuS} g_l V_{CuS} \zeta_{CuS}}{12\pi r^3} \quad (9)$$

where λ_{DDI}^{Al} and λ_{DDI}^{CuS} are the DDI constants of Al and CuS, and the Π_{DDI}^{Al} and Π_{DDI}^{CuS} are the DDI coupling constants of Al and CuS, respectively. We add all three DDI fields into one total DDI field as follows

$$E_{DDI}^{tot} = \Pi_{DDI}^{tot} E_p$$
$$\Pi_{DDI}^{tot} = \Pi_{DDI}^{Au} + \Pi_{DDI}^{Al} + \Pi_{DDI}^{CuS} \quad (10)$$

The total DDI field illuminates all three nanoparticle films.

Note that SPP and DDI fields contain the SPP polarization factor $(\zeta_i, i = Au, Al, CuS)$. We consider that the dielectric constant of the NPs ϵ_m has the following form

$$\epsilon_m = \epsilon_\infty \left(1 - \frac{(\omega_{pl}^m)^2}{\omega_p{}^2 + i\gamma_{pl}^m \omega_p}\right) \quad \text{m=Au, Al, CuS} \quad (11)$$

where ω_{pl}^m is the plasma frequency, γ_{pl}^m is decay rate and ϵ_∞ is the dielectric constant in the large frequency range. Note that the real part of ϵ_m is negative when $\omega_p < \omega_{pl}^m$. When ϵ_m is negative, the denominator of ζ_i can become zero at certain value of the frequency ω_p, which is called the

localized SPP resonance frequency $\omega_p = \omega_{res}^{m}$. It is important to note that Au, Al and CuS NPs exhibit different localized SPP frequencies, denoted as ω_{res}^{Au}, ω_{res}^{Al}, and ω_{res}^{CuS}, respectively.

3.2 Three-coupled Wave Theory for 4HG and 5HG

In this section, we develop a theory for the 4HG and 5HG from Au, Al and CuS NPs. We have shown that there are several electric fields falling on the PNPs, the probe field , three SPP fields (E_{SPP}^{Au}, E_{SPP}^{Al}, and E_{SPP}^{CuS}) and the total DDI field (E_{DDI}^{tot}), thus we use the couple wave theory to calculate the intensities of the 4HG and 5HG emitted by these PNPs.

3.2.1 4HG Field

We assume the probe field with an amplitude E_p, a wavevector k_p and frequency ω_p.This field induces a new 4HG coherent optical wave with the amplitude E_4, wavevector k_4 and frequency of ω_p. The conservation of energy and momentum requires that $\omega_{4p} = 4\omega_p$ and $k_4 = 4k_p$. The 4HG is the four-wave mixing process in a fourth-order nonlinear medium. The main physics behind the 4HG is the interaction among four photons in a fourth-order nonlinear medium. We show the schematic diagram of 4HG in a two-level system in Figure S10a. The probe, SPP and DDI fields produce a 4HG emission field. We have shown in eqns. (4-10) that the probe, SPP and DDI fields have the same frequency, and they are traveling in the same direction. Following the method in the reference[9,10], the fourth-order polarization in the presence of these fields can be written in terms of the fourth-order susceptibility as follows

$$P_m^{(4)} = \epsilon_0 \chi_m^{(4)}(\omega_p, \omega_p, \omega_p, \omega_p)[E_p(\omega_p) + E_{SPP}^{m}(\omega_p) + E_{DDI}^{tot}(\omega_p)]^4 \qquad (12)$$

where subscript m stands for m =Au, Al, CuS and $\chi_m^{(4)}$ is the fourth-order susceptibility of the PNPs.

Note that two SPP fields (E_{SPP}^{Al}, E_{SPP}^{CuS}) are falling on the Au NPs. Similarly, two SPP fields (E_{SPP}^{Au}, E_{SPP}^{CuS}) and two SPP fields (E_{SPP}^{Al}, E_{SPP}^{Au}) are falling on the Al NPs and CuS NPs, respectively. Therefore, when we are calculating 4HG and 5HG, we will combine two SPP fields into one SPP field E_{SPP}^{m}. For example, for Au NPs, we use the notation $E_{SPP}^{m} = E_{SPP}^{CuS} + E_{SPP}^{Al}$. The same notations will be used for Al and CuS NPs.

We consider that all fields are linearly polarized monochromatic plane waves propagating along the z-axis. For simplicity, we consider that all four fields are polarized along x-direction. They can be specifically written as follows

$$E(z) = E_p(z) + E^m_{SPP}(z) + E^{tot}_{DDI}(z) + E^m_4(z) \quad (13)$$

where

$$E_p(z) = A_p(z)e^{-i\omega_p t}e^{ik_p z}$$
$$E^m_{SPP}(z) = A^m_{SPP}(z)e^{-i\omega_p t}e^{ik_p z}$$
$$E^m_{DDI}(z) = A^m_{DDI}(z)e^{-i\omega_p t}e^{ik_p z}$$
$$E^m_4(z) = A^m_4(z)e^{-i\omega_4 t}e^{ik_4 z} \quad (14)$$

where A_p, A_{SPP}, A_{DDI}, and A_4 are the amplitudes of the probe, SPP, DDI and 4HG fields, respectively. The wave vectors can be written in the following form

$$k_p = \frac{2\pi}{\lambda_p} n_1$$
$$k_4 = \frac{2\pi}{\frac{\lambda_p}{4}} n_4 \quad (15)$$

where n_1 and n_4 are refractive indices evaluated at ω_p and $4\omega_p$, respectively and λ_p is the wavelength of the probe field.

We now start with Maxwell's wave equation which includes the nonlinear polarization term $P^{(4)}_{nl}$ as follows

$$\nabla^2 E = \mu_0 \epsilon_0 \frac{\partial^2 E}{\partial t^2} + \mu_0 \frac{\partial^2 P^{(4)}_m}{\partial t^2} \quad (16)$$

where ϵ_0 is the dielectric constant of the vacuum and μ_0 is the magnetic permeability of vacuum. We have considered that the electric fields are polarized along x-direction. We substitute eqns. (13) and (14) into eqn. (16). We use the slowly varying amplitude condition, which is valid in the present case since the size of nanoparticles in our study is much smaller than the wavelength of the probe field. In this way, the amplitude of the probe field is constant over all the nanoparticle domain. This condition is also known as the quasi-static approximation. Using the quasi-static approximation and the method in the literature[9,10], we get the following coupled equations.

$$\frac{dA^m_4}{dz} = +i\frac{k_4 \epsilon_0}{2n_4^2}\chi^{(4)}_m (A_p(z) + A^m_{SPP}(z) + A^{tot}_{DDI}(z))^4 e^{i\Delta k_4 z} \quad (17)$$

where subscript m stands for m =Au, Al, CuS. Here Δk_4 is the phase mismatch factor and written as $\Delta k_4 = 4k_p - k_4 = 8\pi(n_1 - n_4)/\lambda_p$. Note that phase factor is proportional to the refractive-index difference at frequency ω_p and $4\omega_p$. In general, the refractive index of a medium is always a function of the probe frequency due to the dispersion effect.

Inserting the expression of the SPP and DDI fields from eqns. (4-9) into eqn. (17), we get

$$\frac{dA_4^m}{dz} = +i\frac{k_4\epsilon_0}{2n_4^2}(\Lambda_{sd}^m)^4\chi_m^{(4)}A_p^4(z)e^{i\Delta k_4 z} \tag{18}$$

where

$$\Lambda_{sd}^m = 1 + \Pi_{SPP}^m + \Pi_{DDI}^{tot} \tag{19}$$

Now, let us solve eqn. (18). We consider the initial condition: at z = 0, $A_p(z) = A_p(0)$ and $A_4(z=0) = 0$. Here $A_p(0)$ is the initial amplitude of the incident probe wave at the incident surface of the PNPs. Under this condition, the solution of eqn. (18) leads to the following solution at z = L

$$A_4^m(L) = \frac{k_4\epsilon_0 L}{2n_4^2}(\Lambda_{sd}^m)^4\chi_m^{(4)}A_p^4(0)F(\Delta k_4 L) \tag{20}$$

where

$$F(\Delta k_4 L) = e^{i\Delta k_4 L/2}\frac{\sin(\Delta k_4 L/2)}{\Delta k_4 L/2} \tag{21}$$

where $A_4(L)$ is the amplitude of the fourth harmonic wave leaving the PNPs while $F(\Delta k_4 L)$ is called the phase factor. We assume that energy and momentum are conserved in 4HG process. This means, $\omega_4 = 4\omega_p$ and $\Delta k = 0$. In this case, the phase function reduces to

$$\lim_{\Delta k_4 \to 0} F(\Delta k_4 L) = \lim_{\Delta k_4 \to 0} e^{i\Delta k_4 L/2}\frac{\sin(\Delta k_4 L/2)}{\Delta k_4 L/2} = 1 \tag{22}$$

Inserting eqn. (22) into eqn. (20) and we get

$$A_4^m(L) = \frac{k_4\epsilon_0 L}{2n_4^2}(\Lambda_{sd}^m)^4\chi_m^{(4)}A_p^4(0) \tag{23}$$

3.2.2 5HG Field

The probe field induces a new 5HG coherent optical wave with amplitude E_5, wavevector k_5 and frequency ω_5. Conservation of energy and momentum requires that $\omega_5 = 5\omega_p$ and $k_5 = 5k_p$. The main physics behind the 5HG is the interaction among five photons in a fifth-order nonlinear medium. The Figure S10b shows the schematic diagram of 5HG in a two-level system. The fifth-

order polarization in the presence of the probe, SPP and DDI fields can be written in terms of the fifth-order susceptibility as follows

$$P_m^{(5)} = \epsilon_0\, \chi_m^{(5)}(\omega_p, \omega_p, \omega_p, \omega_p, \omega_p)\left[E_p(\omega_p) + E_{SPP}^m(\omega_p) + E_{DDI}^{tot}(\omega_p)\right]^5 \quad (24)$$

where $\chi_m^{(5)}$ is the fifth-order susceptibility of the PNPs.

We consider that all fields are fields are propagating along the *z*-axis and polarized along x-direction. They can be specifically written as follows

$$E(z) = E_p(z) + E_{SPP}^m(z) + E_{DDI}^{tot}(z) + E_5^m(z) \quad (25)$$

where

$$E_p(z) = A_p(z)e^{-i\omega_p t}e^{ik_p z}$$

$$E_{SPP}^m(z) = A_{SPP}^m(z)e^{-i\omega_p t}e^{ik_p z}$$

$$E_{DDI}^m(z) = A_{DDI}^m(z)e^{-i\omega_p t}e^{ik_p z}$$

$$E_5^m(z) = A_5^m(z)e^{-i\omega_5 t}e^{ik_5 z} \quad (26)$$

where A_p, A_{SPP}, A_{DDI}, and A_5 are the amplitudes of the probe, SPP, DDI and 5HG fields, respectively. The wave vectors can be written in the following form

$$k_p = \frac{2\pi}{\lambda_p} n_1,\ k_5 = \frac{2\pi}{\frac{\lambda_p}{5}} n_5 \quad (27)$$

where n_1 and n_5 are refractive indices evaluated at ω_p and $5\omega_p$, respectively.

We substitute eqns. (24) and (25) into eqn. (16) and apply the quasi-static approximation. We obtain the following coupled equation:

$$\frac{dA_5^m}{dz} = +i\frac{k_5\epsilon_0}{2n_5^2}(\Lambda_{sd}^m)^5\chi_m^{(5)}A_p^5(z)e^{i\Delta k_5 z} \quad (28)$$

where Δk_5 is the phase mismatch factor and written as $\Delta k_5 = 5k_p - k_5 = 10\pi(n_1 - n_5)/\lambda_p$. Note the phase factor is proportional to the refractive-index difference at frequency ω_p and $5\omega_p$.

Now, let us solve eqn. (28). We consider the similar initial condition: at z = 0, $A_p(z) = A_p(0)$ and $A_5(z=0) = 0$. Here $A_p(0)$ is the initial amplitude of the incident probe wave at the incident surface of the PNPs. Under this condition, the solution of eqn. (28) leads to the following solution at z = L

$$A_5^m = \frac{k_5 \epsilon_0 L}{2n_5^2} (\Lambda_{sd}^m)^5 \chi_m^{(5)} A_p^5(0) F(\Delta k_5 L) \quad (29)$$

where

$$F(\Delta k_5 L) = e^{i\Delta k_5 L/2} \frac{\sin(\Delta k_5 L/2)}{\Delta k_5 L/2} \quad (30)$$

where $A_5(L)$ is the amplitude of the fifth harmonic wave leaving the PNPs. Here $F(\Delta k_5 L)$ is the mismatching phase factor. In the phase matching condition, eqn. (29) reduces as follow

$$A_5^m = \frac{k_5 \epsilon_0 L}{2n_5^2} (\Lambda_{sd}^m)^5 \chi_{Al}^{(5)} A_p^5(0) \quad (31)$$

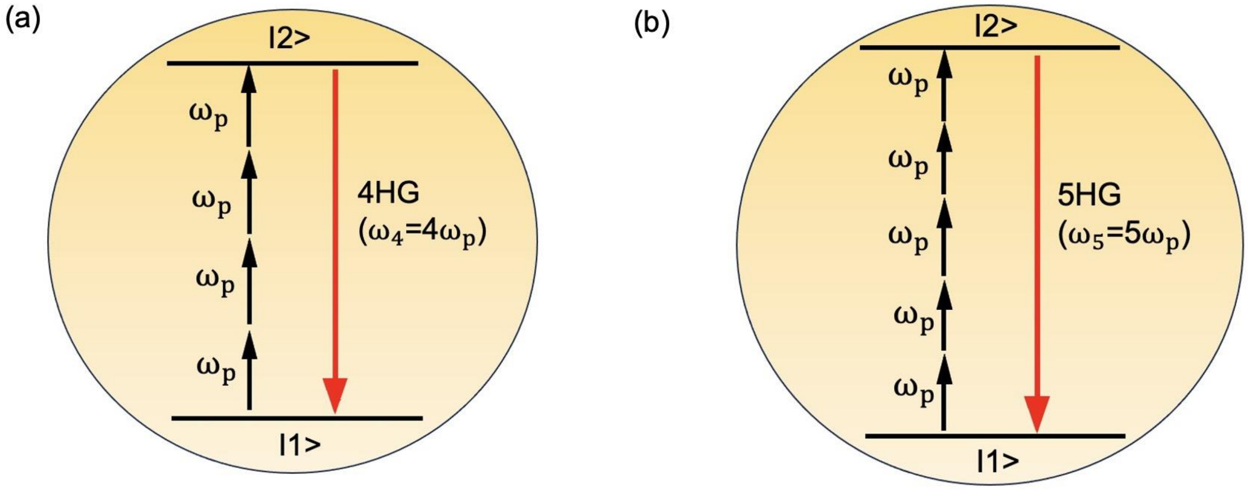

Figure S10. A schematic diagram for the 4HG and 5HG in a two-level plasmonic system. Energy levels are denoted as |1> and |2>.

3.3 Susceptibility for 4HG and 5HG with Density Matrix Method

In this section, we calculate the susceptibilities $\chi_m^{(4)}$ and $\chi_m^{(5)}$ appearing in eqn. (12) and (24) using the density matrix method. These susceptibilities are responsible for the 4HG and 5HG emission in the Au, Al and CuS NPs. We have established that these PNPs have a localized SPP resonance frequencies ω_m^{res} $(m = Au, Al, CuS)$. Therefore, we treat them as a two-level system whose ground state and excited state are denoted as |1> and |2>, respectively. The frequency difference between levels |1> and |2> is expressed as ω_{res}^m. This model was used recently in reference[11].

3.3.1 $\chi_m^{(4)}$ and $\chi_m^{(5)}$ of Au NPs

As an example, we first calculate the susceptibility for Au NPs. We study the effect of CuS and Al NPs on the 4HG and 5HG emission of Au NPs. The susceptibilities of the Au are related to the density of matrix element as follows[12,13]

$$\chi_{Au}^{(4)} = \frac{2\mu_{21}(\rho_{Au}^{(4)})}{\epsilon_0 \, V_{Au}(E_p)^4}$$

$$\chi_{Au}^{(5)} = \frac{2\mu_{21}(\rho_{Au}^{(5)})}{\epsilon_0 V_{Au}(E_p)^5} \quad (32)$$

where $\rho_{Au}^{(4)} = \rho_{21}^{(4)}$ and $\rho_{Au}^{(5)} = \rho_{21}^{(5)}$ are the matrix elements for density matrix between states |1> and |2>. To calculate the density matrix element, we need to figure out how many electric fields are falling on the Au nanoparticle. There are four fields interacting with Au NPs: the probe field with amplitude E_p, two SPP fields produced by Al and CuS NPs (E_{SPP}^{CuS}, E_{SPP}^{Al}) and the total DDI field. Using the dipole and rotating wave approximation[12-14], the interaction Hamiltonian is found as follows

$$H_{Au} = \hbar\Omega_p\sigma_{21} + \hbar\Omega_{SPP}^{CuS}\sigma_{21} + \hbar\Omega_{SPP}^{Al}\sigma_{21} + \hbar\Omega_{DDI}^{tot}\sigma_{21} + h.c. \quad (33)$$

where

$$\Omega_P = \frac{\mu_{21}E_p}{\hbar}$$

$$\Omega_{SPP}^{CuS} = \frac{\mu_{21}E_{SPP}^{CuS}}{\hbar}$$

$$\Omega_{SPP}^{Al} = \frac{\mu_{21}E_{SPP}^{Al}}{\hbar}$$

$$\Omega_{DDI}^{tot} = \frac{\mu_{21}E_{DDI}^{tot}}{\hbar} \quad (34)$$

where h.c. stands for the Hermitian conjugate. Here $\sigma_{21} = |2 >< |1|$ is the SPP polariton creation operator. The parameter Ω_P, Ω_{SPP} and Ω_{DDI} are called the Rabi frequencies associated with the probe field, SPP field and DDI field, respectively. The first term in the Hamiltonian is the polariton and photon interaction. The second and third terms are the interaction between the Au NPs with the SPP fields produced by the CuS NPs and Al NPs, respectively. The last term is the interaction between Au NP and the total DDI field.

Following the method of references[12-14] and with the help of Hamiltonian eqn. (33), the equations

of the motion for density matrix elements are found as follows

$$\frac{d\rho_{22}}{dt} = -(\gamma_{21})\rho_{22} - i\Lambda_{sd}^{Au}\Omega_p(\rho_{12} - \rho_{21})$$

$$\frac{d\rho_{21}}{dt} = -(d_{21})\rho_{21} + i\Lambda_{sd}^{Au}\Omega_p(\rho_{22} - \rho_{11}) \quad (35)$$

where

$$\Lambda_{sd}^{Au} = 1 + \Pi_{SPP}^{CuS} + \Pi_{SPP}^{Al} + \Pi_{DDI}^{tot}$$

$$d_{21} = \delta_p + i\gamma_{21}$$

$$\delta_p = \omega_{Au}^{res} - n\omega_p \text{ (n=4 for 4HG and n=5 for 5HG)} \quad (36)$$

where δ_p is called the field detuning. Physical quantity γ_{21} is the exciton decay rates for transition $|2\rangle \leftrightarrow |1\rangle$. Note that we use notation $\rho_{21}^{(4)} = \rho_{Au}^{(4)}$. The density matrices ρ_{11} and ρ_{22} satisfy $\rho_{11} + \rho_{22} = 1$. The 4HG and 5HG density matrix element are calculated in the form of $(\Lambda_{sd}^{Au}\Omega_p)^4$ and $(\Lambda_{sd}^{Au}\Omega_p)^5$ by solving eqn. (35) in the steady sate. After some mathematical manipulations we obtain analytical expressions for $\rho_{Au}^{(4)}$ and $\rho_{Au}^{(5)}$as follows

$$\rho_{Au}^{(4)} = \frac{(\Lambda_{sd}^{Au}\Omega_p)^4}{\gamma_{21}^4} F_{Au}^{(4)}$$

$$F_{Au}^{(4)} = \frac{4i\gamma_{21}^3(d_{21}+d_{21}^*)^2}{d_{21}(d_{21}d_{21}^*)^2} \quad (37)$$

$$\rho_{Au}^{(5)} = \frac{(\Lambda_{sd}^{Au}\Omega_p)^5}{\gamma_{21}^5} F_{Au}^{(5)}$$

$$F_{Au}^{(5)} = -\frac{4i\gamma_{21}^4(d_{21}+d_{21}^*)^2}{d_{21}(d_{21}d_{21}^*)^2} \quad (38)$$

Note that $\rho_{Au}^{(4)}$, $\rho_{Au}^{(5)}$, $F_{Au}^{(4)}$ and $F_{Au}^{(5)}$ are all unitless quantities. We insert eqn. (37,38) for density matrices $\rho_{Au}^{(4)}$ and $\rho_{Au}^{(5)}$ into expression of the susceptibilities in eqn. (32), and get

$$\chi_{Au}^{(4)} = \frac{32\mu_{21}^5(\Lambda_{sd}^{Au})^4}{\epsilon_0 V_{Au}\hbar^4\gamma_{21}{}^4} F_{Au}^{(4)}$$

$$\chi_{Au}^{(5)} = \frac{64\mu_{21}^6(\Lambda_{sd}^{Au})^5}{\epsilon_0 V_{Au}\hbar^5\gamma_{21}{}^5} F_{Au}^{(5)} \quad (39)$$

3.3.2 The susceptibilities for Al and CuS NPs

Following the above method of Au NPs, we have also calculated the susceptibilities for the Al and

CuS NPs as follows

$$\chi_{Al}^{(4)} = \frac{32\mu_{21}^{5}\left(\Lambda_{sd}^{Al}\right)^{4}}{\epsilon_0 V_{Al}\hbar^4{\gamma_{21}}^4} F_{Al}^{(4)}$$

$$\chi_{Al}^{(5)} = \frac{64\mu_{21}^{6}\left(\Lambda_{sd}^{Al}\right)^{5}}{\epsilon_0 V_{Al}\hbar^5{\gamma_{21}}^5} F_{Al}^{(5)} \tag{40}$$

$$\chi_{CuS}^{(4)} = \frac{32\mu_{21}^{5}\left(\Lambda_{sd}^{CuS}\right)^{4}}{\epsilon_0 V_{CuS}\hbar^4{\gamma_{21}}^4} F_{CuS}^{(4)}$$

$$\chi_{CuS}^{(5)} = \frac{64\mu_{21}^{6}\left(\Lambda_{sd}^{CuS}\right)^{5}}{\epsilon_0 V_{CuS}\hbar^5{\gamma_{21}}^5} F_{CuS}^{(5)} \tag{41}$$

3.4 The 4HG and 5HG Intensities

In this section we calculate the intensities of 4HG and 5HG for Au, Al and CuS NPs.

3.4.1 4HG intensities for PNPs

Let us calculate the 4HG intensity for the Au NPs first. The expression of the 4HG intensity can be calculated as follows[9,10]

$$I_{4HG}^{Au} = \frac{1}{2}\sqrt{\frac{\epsilon_0}{\mu_0}} n_4 \left|A_4^{Au}\right|^2 \tag{42}$$

where A_4^{Au} is the amplitude of 4HG field due to the Au nanoparticle. Inserting eqn. (20) into eqn. (42), we get the expression of 4HG intensity

$$I_{4HG}^{Au} = I_p^4 \left(\frac{2{k_4}^2\mu_0^2L^2}{n_4^3}\right)\left(\Lambda_{sd}^{Au}\right)^8 \left|\chi_{Au}^{(4)}\right|^2 |F(\Delta k_4 L)|^2 \tag{43}$$

where

$$I_p = \frac{1}{2}\sqrt{\frac{\epsilon_0}{\mu_0}} \left|A_p(0)\right|^2 \tag{44}$$

Inserting the expression of $\chi_{Au}^{(4)}$ from eqn. (39) into eqn. (43), we find the final expression of the intensity of the 4HG for Au NPs as follows

$$I_{4HG}^{Au} = I_p^4 \alpha_4^{Au}\left(\Lambda_{sd}^{Au}\right)^{16} \left|F_{Au}^{(4)}\right|^2 |F(\Delta k_4 L)|^2 \tag{45}$$

$$\alpha_4^{Au} = \frac{\alpha_4^0}{V_{Au}^2}$$

$$\alpha_4^0 = \left(\frac{8n_4\mu_0^{\frac{3}{2}}}{\epsilon_0^{\frac{3}{2}}}\right)\left(\frac{16\mu_{21}^5 k_4 L}{n_4^2 \hbar^4 \gamma_{21}{}^4}\right)^2 \tag{46}$$

One can see that the intensity of the fourth harmonic is proportional to the fourth power of the intensity of the incident probe field. It also varies periodically along the *z*-axis due to the phase mismatch and the period of the intensity variation is $\Delta k_4 L/2$. Note that a larger Δk_4 leads to a smaller peak intensity and more rapid variation.

Similarly, following the method of Au NPs, we can get 4HG intensities for Al and CuS NP.

$$I_{4HG}^{Al} = I_p^4 \alpha_4^{Al} \left(\Lambda_{sd}^{Al}\right)^{16} \left|F_{Al}^{(4)}\right|^2 |F(\Delta k_4 L)|^2$$

$$I_{4HG}^{CuS} = I_p^4 \alpha_4^{CuS} \left(\Lambda_{sd}^{CuS}\right)^{16} \left|F_{CuS}^{(4)}\right|^2 |F(\Delta k_4 L)|^2 \tag{47}$$

where

$$\alpha_4^{Al} = \frac{\alpha_4^0}{V_{Al}^2}$$

$$\alpha_4^{CuS} = \frac{\alpha_4^0}{V_{CuS}^2} \tag{48}$$

3.4.2 5HG intensities for PNPs

Following the above method of the 4HG, we can calculate the intensity of the 5HG for Au, Al and CuS NPs as follows

$$I_{5HG}^{Au} = I_p^5 \alpha_5^{Au} \left(\Lambda_{sd}^{Au}\right)^{20} \left|F_{Au}^{(5)}\right|^2 |F(\Delta k_5 L)|^2$$

$$I_{5HG}^{Al} = I_p^5 \alpha_5^{Al} \left(\Lambda_{sd}^{Al}\right)^{20} \left|F_{Al}^{(5)}\right|^2 |F(\Delta k_5 L)|^2$$

$$I_{5HG}^{CuS} = I_p^5 \alpha_5^{CuS} \left(\Lambda_{sd}^{CuS}\right)^{20} \left|F_{CuS}^{(5)}\right|^2 |F(\Delta k_5 L)|^2 \tag{49}$$

where

$$\alpha_5^{Au} = \frac{\alpha_5^0}{V_{Au}^2}$$

$$\alpha_5^{Al} = \frac{\alpha_5^0}{V_{Al}^2}$$

$$\alpha_5^{CuS} = \frac{\alpha_5^0}{V_{CuS}^2}$$

$$\alpha_5^0 = \left(\frac{16 n_4 \mu_0^2}{\epsilon_0^2}\right)\left(\frac{32 \mu_{21}^6 k_5 L}{n_5^2 \hbar^5 {\gamma_{21}}^5}\right)^2 \tag{50}$$

3.5 4HG and 5HG intensities for the Al/Au/CuS heterostructure

From the previous section, we obtain the expressions of 4HG and 5HG intensities from each film with the presence of SPP fields and total DDI fields. Then the far-field intensity of the 4HG and 5HG output by the heterostructure will be the sum of the output signal from each component

$$I_{4HG}^{AlAuCuS} = I_{4HG}^{Al} + I_{4HG}^{Au} + I_{4HG}^{CuS} \tag{51}$$

$$I_{5HG}^{AlAuCuS} = I_{5HG}^{Al} + I_{5HG}^{Au} + I_{5HG}^{CuS} \tag{52}$$

As a result, under the phase matching condition, the final statement of the 4HG and 5HG intensity in Al/Au/CuS films will be written as

$$I_{4HG}^{AlAuCuS} = I_p^4 \left\{\alpha_4^{Al}\left(\Lambda_{sd}^{Al}\right)^{16}\left|F_{Al}^{(4)}\right|^2 + \alpha_4^{Au}\left(\Lambda_{sd}^{Au}\right)^{16}\left|F_{Au}^{(4)}\right|^2 + \alpha_4^{CuS}\left(\Lambda_{sd}^{CuS}\right)^{16}\left|F_{CuS}^{(4)}\right|^2\right\} \tag{53}$$

$$I_{5HG}^{AlAuCuS} = I_p^5 \left\{\alpha_5^{Al}\left(\Lambda_{sd}^{Al}\right)^{20}\left|F_{Al}^{(5)}\right|^2 + \alpha_5^{Au}\left(\Lambda_{sd}^{Au}\right)^{20}\left|F_{Au}^{(5)}\right|^2 + \alpha_5^{CuS}\left(\Lambda_{sd}^{CuS}\right)^{20}\left|F_{CuS}^{(5)}\right|^2\right\} \tag{54}$$

3.6 4HG intensities for the Al/CuS heterostructure

Following the similar procedure, the 4HG intensity in hybrid Al/CuS films can be also expressed by introducing the SPP and DDI electric field from the coupling of Al and CuS plasmons:

$$I_{4HG}^{AlCuS} = I_{4HG}^{Al} + I_{4HG}^{CuS} \tag{55}$$

$$I_{4HG}^{AlCuS} = I_p^4 \left\{\alpha_4^{Al}\left(\Lambda_{sd}^{Al}\right)^{16}\left|F_{Al}^{(4)}\right|^2 + \alpha_4^{CuS}\left(\Lambda_{sd}^{CuS}\right)^{16}\left|F_{CuS}^{(4)}\right|^2\right\} \tag{56}$$

where

$$\Lambda_{sd}^{Al} = 1 + \Pi_{SPP}^{CuS} + \Pi_{DDI}^{tot} \tag{57}$$

$$\Lambda_{sd}^{CuS} = 1 + \Pi_{SPP}^{Al} + \Pi_{DDI}^{tot} \tag{58}$$

$$\Pi_{DDI}^{tot} = \Pi_{DDI}^{Al} + \Pi_{DDI}^{CuS} \tag{59}$$

3.7 Comparison of theory and experiments

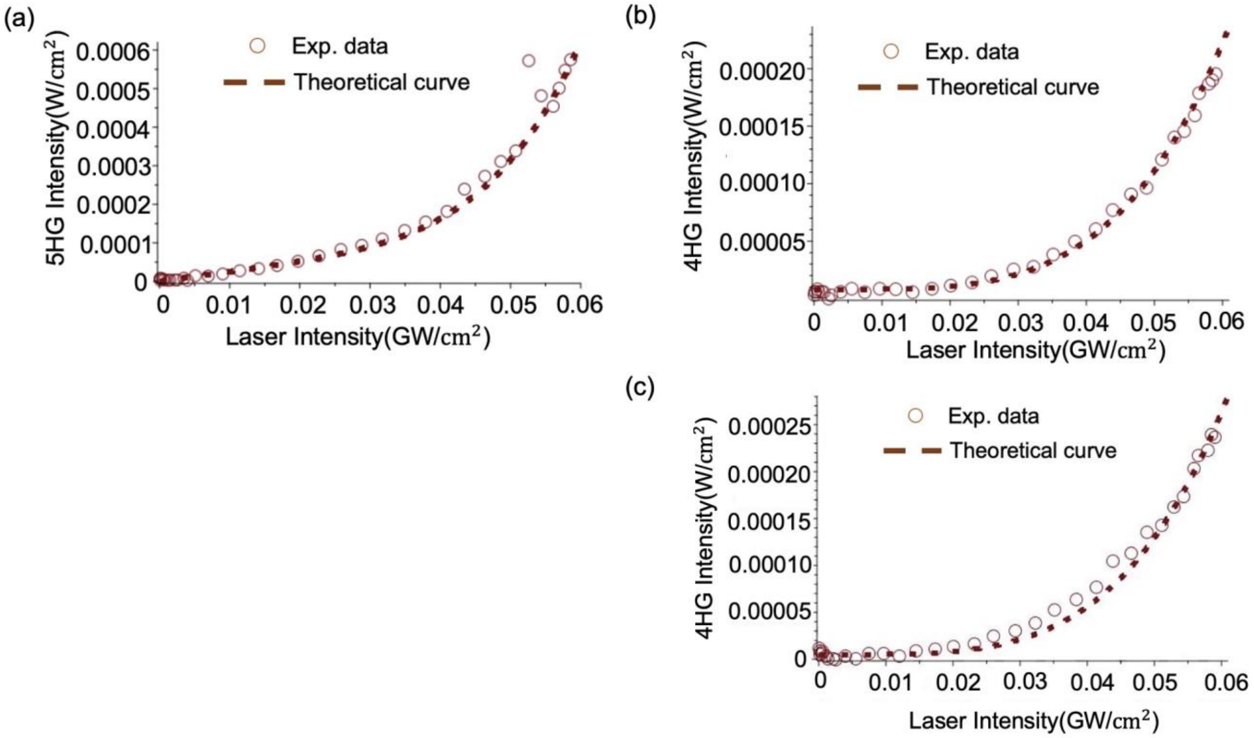

Figure S11. A comparison between the theory and experiments for the intensity of harmonic generation signal as a function of the laser intensity. (a) 5HG and (b) 4HG from Al/Au/CuS films. (c) 4HG from Al/CuS films. The experimental points are shown in open circles and the dashed line corresponds to the theoretical fitting derived from Equation 53, 54 and 56, respectively.

.

The Figure S11 shows a fairly good agreement between the experimental results and theoretical calculation. Here, the DDI coupling constants and SPP coupling constant values result from the fitting procedure. The linear least square fitting method of Matlab (Curve Fitting Toolbox 23.3) is used for the theoretical fitting. In Al/Au/CuS films, $\Pi_{\mathrm{SPP}}^{\mathrm{m}}$=$\Pi_{\mathrm{DDI}}^{\mathrm{m}}$=2.64. The R^2 values of the fitting are 0.991 and 0.971 for 4HG and 5HG, respectively; in Al/CuS films, $\Pi_{\mathrm{SPP}}^{\mathrm{Al}} = 2.26$, $\Pi_{\mathrm{SPP}}^{\mathrm{CuS}} = 1.53$, and $\Pi_{\mathrm{DDI}}^{\mathrm{m}} = 1.79$. The R^2 value of the fitting is 0.988. It is noteworthy that our theoretical predictions demonstrate excellent concordance with experimental observations, validating the efficacy of our model.

Supplementary References

[1] Johnson, P. B.; Christy, R. W. Optical Constant of the Nobel Metals. *Phys Rev B* **1972**, *6* (12), 4370–4379.

[2] Blaber, M. G.; Arnold, M. D.; Fold, M. J. Search for the Ideal Plasmonic Nanoshell: the Effect of Surface Scattering and Alternatives to Gold and Silver

[3] Xie, Y.; Carbone, L.; Nobile, C.; Grillo, V.; D′Agostino, S.; Sala, F. D.; Giannini, C.; Altamura, D.; Oelsner, C.; Kryschi, C.; Cozzoli, P. D. Metallic-like stoichiometric copper sulfide nanocrystals: Phase- and shape-selective synthesis, near-infrared surface plasmon resonance properties, and their modeling. *ACS Nano,* ***2013,*** *7,* 7252.

[4] Motl, N. E.; Ewusi-Annan, E.; Sines, I. T.; Jensen, L.; Schaak, R. E. Au-Cu alloy nanoparticles with tunable compositions and plasmonic properties: Experimental determination of composition and correlation with theory. *J Phys Chem C,* ***2010***, *114*, 19623.

[5] McClain, M. J.; Schlather, A. E.; Ringe, E.; King, N. S.; Liu, L.; Manjavacas, A.; Knight, M. W.; Kumar, I.; Whitmire, K. H.; Everitt, H. O.; Nordlander, P.; Halas, N. J. Aluminum Nanocrystals. *Nano Lett* **2015**, *15*, 2751.

[6] Singh, M. R.; Black, K. Anomalous Dipole–Dipole Interaction in an Ensemble of Quantum Emitters and Metallic Nanoparticle Hybrids. *J Phys Chem C* **2018**, *122*, 26584.

[7] Novotny, L.; Hecht, B. Principle of Nano-optics, Cambridge University Press, Cambridge, **2006**.

[8] Sarid, D.; Challener, W. A. Modern Introduction to Surface Plasmons: Theory, Mathematica Modeling, and Applications, Cambridge University Press, Cambridge, **2010**.

[9] He, G. S. Nonlinear Optics and Photonics, Oxford University Press, Oxford, **2015**.

[10] Yariv, A.; Yeh, P. Photonics: Optical Electronics in Modern Communications (6th Edition), Oxford University Press, **2007**.

[11] Spear, N. J.; Yan, Y.; Queen, J. M.; Singh, M. R.; Macdonald, J. E.; Haglund, R. F. Surface plasmon mediated harmonically resonant effects on third harmonic generation from Au and CuS nanoparticle films. *Nanophotonics* **2023**, *12*, 273.

[12] Hamura, E.; Kawabe, Y.; Yamanaka, A. Quantum Nonlinear Optics, Springer, Tokyo, **2007**.

[13] Scully, M. O.; Zubairy, M. S. Quantum Optics, Cambridge University Press, London, **1997**.

[14] Singh, M. R. Electronic, Photonic, Polaritonic and Plasmonic Materials, Wiley Custom,

Toronto, **2014**.
